# Functional diversity of subgroup 5 R2R3-MYBs promoting proanthocyanidin biosynthesis and their key residues and motifs in tea plant

**DOI:** 10.1093/hr/uhad135

**Published:** 2023-07-05

**Authors:** Tianming Jiao, Yipeng Huang, Ying-Ling Wu, Ting Jiang, Tongtong Li, Yanzhuo Liu, Yvchen Liu, Yunyun Han, Yajun Liu, Xiaolan Jiang, Liping Gao, Tao Xia

**Affiliations:** State Key Laboratory of Tea Plant Biology and Utilization/Key Laboratory of Tea Biology and Tea Processing of Ministry of Agriculture/Anhui Provincial Laboratory of Tea Plant Biology and Utilization, Anhui Agricultural University, West 130 Changjiang Road, Hefei 230036 Anhui, China; State Key Laboratory of Tea Plant Biology and Utilization/Key Laboratory of Tea Biology and Tea Processing of Ministry of Agriculture/Anhui Provincial Laboratory of Tea Plant Biology and Utilization, Anhui Agricultural University, West 130 Changjiang Road, Hefei 230036 Anhui, China; School of Life Science, Anhui Agricultural University, Hefei 230036, Anhui, China; School of Life Science, Anhui Agricultural University, Hefei 230036, Anhui, China; State Key Laboratory of Tea Plant Biology and Utilization/Key Laboratory of Tea Biology and Tea Processing of Ministry of Agriculture/Anhui Provincial Laboratory of Tea Plant Biology and Utilization, Anhui Agricultural University, West 130 Changjiang Road, Hefei 230036 Anhui, China; State Key Laboratory of Tea Plant Biology and Utilization/Key Laboratory of Tea Biology and Tea Processing of Ministry of Agriculture/Anhui Provincial Laboratory of Tea Plant Biology and Utilization, Anhui Agricultural University, West 130 Changjiang Road, Hefei 230036 Anhui, China; School of Life Science, Anhui Agricultural University, Hefei 230036, Anhui, China; School of Life Science, Anhui Agricultural University, Hefei 230036, Anhui, China; School of Life Science, Anhui Agricultural University, Hefei 230036, Anhui, China; State Key Laboratory of Tea Plant Biology and Utilization/Key Laboratory of Tea Biology and Tea Processing of Ministry of Agriculture/Anhui Provincial Laboratory of Tea Plant Biology and Utilization, Anhui Agricultural University, West 130 Changjiang Road, Hefei 230036 Anhui, China; School of Life Science, Anhui Agricultural University, Hefei 230036, Anhui, China; State Key Laboratory of Tea Plant Biology and Utilization/Key Laboratory of Tea Biology and Tea Processing of Ministry of Agriculture/Anhui Provincial Laboratory of Tea Plant Biology and Utilization, Anhui Agricultural University, West 130 Changjiang Road, Hefei 230036 Anhui, China

## Abstract

The tea plant (*Camellia sinensis*) is rich in polyphenolic compounds. Particularly, flavan-3-ols and proanthocyanidins (PAs) are essential for the flavor and disease-resistance property of tea leaves. The fifth subgroup of R2R3-MYB transcription factors comprises the primary activators of PA biosynthesis. This study showed that subgroup 5 R2R3-MYBs in tea plants contained at least nine genes belonging to the TT2, MYB5, and MYBPA types. Tannin-rich plants showed an expansion in the number of subgroup 5 R2R3-MYB genes compared with other dicotyledonous and monocot plants. The MYBPA-type genes of tea plant were slightly expanded. qRT–PCR analysis and GUS staining analysis of promoter activity under a series of treatments revealed the differential responses of CsMYB5s to biotic and abiotic stresses. In particular, *CsMYB5a*, *CsMYB5b*, and *CsMYB5e* responded to high-intensity light, high temperature, MeJA, and mechanical wounding, whereas *CsMYB5f* and *CsMYB5g* were only induced by wounding. Three genetic transformation systems (*C. sinensis*, *Nicotiana tabacum*, and *Arabidopsis thaliana*) were used to verify the biological function of CsMYB5s. The results show that *CsMYB5a*, *CsMYB5b*, and *CsMYB5e* could promote the gene expression of *CsLAR* and *CsANR.* However, *CsMYB5f* and *CsMYB5g* could only upregulate the gene expression of *CsLAR* but not *CsANR.* A series of site-directed mutation and domain-swapping experiments were used to verify functional domains and key amino acids of CsMYB5s responsible for the regulation of PA biosynthesis. This study aimed to provide insight into the induced expression and functional diversity model of PA biosynthesis regulation in tea plants.

## Introduction

Proanthocyanidins (PAs), which are products of the condensation of flavan-3-ol units, are widely present in the plant kingdom [1, 2]. The PAs are the most abundant polyphenol compounds in the tea plant (*Camellia sinensis*). They include flavan-3-ols or catechin monomers [epicatechin (EC), catechin (C), epigallocatechin (EGC), gallocatechin (GC), epicatechin gallate (ECG), epigallocatechin gallate (EGCG)] and their polymers [3]. EGCG has been shown to have important physiological functions in the human body owing to its antimicrobial, antipathogenic, antioxidant, and protein-binding properties [4, 5]. Due to the protein-binding and metal ion-chelating capacity of PAs, they are likely to be potentially resistant compounds. For example, recently it was found that PAs could bind Al^3+^ in tea plants, indicating their involvement in reducing the toxicity of Al^3+^ to plants [6].

The PA biosynthesis pathway and regulatory network have been well studied in plants [7]. Studies have shown that PA biosynthesis is regulated by the fifth subgroup of the R2R3-MYB transcription factor (TF) family. Subgroup 5 R2R3-MYB was further grouped based on phylogenetic tree analysis, C-terminal conserved motifs, and biological functions. Early research suggested that subgroup 5 R2R3-MYBs could be divided into two clades, including TT2-type and MYB5-type activators in tea plant [8]. All members of the TT2-type clade contain a TT2-box, whereas the MYB5-type clade contains two conserved motifs called motif C1 and motif C3 at the C terminus. However, recent studies have found that MYB5-type members in some species contain motif G-28 instead of motif C3, such as *DkMYB4* [9], *VvMYBPA1* [10], *CsMYB5e* [8], and so on. Hence, subgroup 5 R2R3-MYBs in plants were divided into three clades: TT2 type, MYB5 type, and MYBPA type.

The three types of subgroup 5 R2R3-MYBs were functionally different. The TT2 types have been reported to play an important role in regulating PA biosynthesis by regulating LAR and ANR, such as *PtMYB134* [11], *MtMYB14* [12], *TaMYB14* [13], *RrMYB10* [14], and *AtTT2* [15]. On the other hand, the functions of MYB5 type are diverse in different species, such as regulation of the biosynthesis of anthocyanins and PA in *Vitis vinifera* [16], promotion of the biosynthesis of seed mucilage in *Medicago truncatula* [12], regulation of epidermal cell growth in leaves of *Arabidopsis thaliana* [17], and regulation of vacuole pH in *Petunia hybrida* [18]. The function of the MYBPA type was first identified in *V. vinifera*, suggesting that it may be specific to the PA pathway by promoting the expression of *VvLAR* and *VvANR* [10]. Subsequently, *DkMYB4* in *Diospyros kaki* [9], *PtMYB115* in *Populus tremula* [19], and *VmMYBPA1* in *Vaccinium myrtillus* [20] were functionally verified. In particular, these plants belong to tannin-rich species.

Biotic and abiotic stresses can regulate PA biosynthesis by regulating the gene expression of subgroup 5 R2R3-MYBs in plants. For example, mechanical wounding, UV-B, and pathogen infection promoted the expression of *PtMYB134* in poplar [11]; salicylic acid could induce the expression of MYB-bHLH-WD40 in poplar to inhibit rust damage [21]; the expression of *DkMYB4* in persimmons was induced by low temperatures [22]; and reactive oxygen strongly induced the expression of *RrMYB5* and *RrMYB10* in *Rosa rugosa* to effectively clear reactive oxygen [14].

The biosynthetic pathways and regulatory networks of PAs in tea plants have been intensively studied for a long time. *CsMYB5a*, *CsMYB5b*, and *CsMYB5e* belong to subgroup 5 R2R3-MYBs in tea plants and have been reported to promote the biosynthesis of catechins and PAs in transgenic tobacco. However, no MYB5-type members homologous to *AtMYB5* have been identified. Therefore, it is necessary to fully identify CsMYB5s in the tea plant and to verify their functions. At the same time, a comprehensive analysis of the responses of the three types of MYB5s to different biotic and abiotic stresses in tea plants is important.

In this study, we found that subgroup 5 R2R3-MYBs in tea plants contained at least nine genes belonging to types TT2, MYB5, and MYBPA. *CsMYB5f* and *CsMYB5g* are reported for the first time. Differences in PA accumulation in CsMYB5s transiently transgenic *C. sinensis* and stable transgenic *Nicotiana tabacum* and *A. thaliana* indicate the functional diversity of the CsMYB5 family. A series of site-directed mutation and domain-swapping experiments led to the identification of key amino acids and functional domains that are responsible for the regulation of PA biosynthesis. Tissue-specific expression and differential expression under various biotic and abiotic stresses suggest that the physiological functions of CsMYB5s are diverse. In particular, *CsMYB5a*, *CsMYB5b*, and *CsMYB5e* responded to high-intensity light, high temperature, JA, and mechanical wounding, whereas *CsMYB5f* and *CsMYB5g* were only induced by mechanical wounding. This study deepens our understanding of the complexities involved in the regulation of PA biosynthesis in tea plant.

## Results

### Subgroup 5 R2R3-MYBs in tea plants

**Figure 1:**
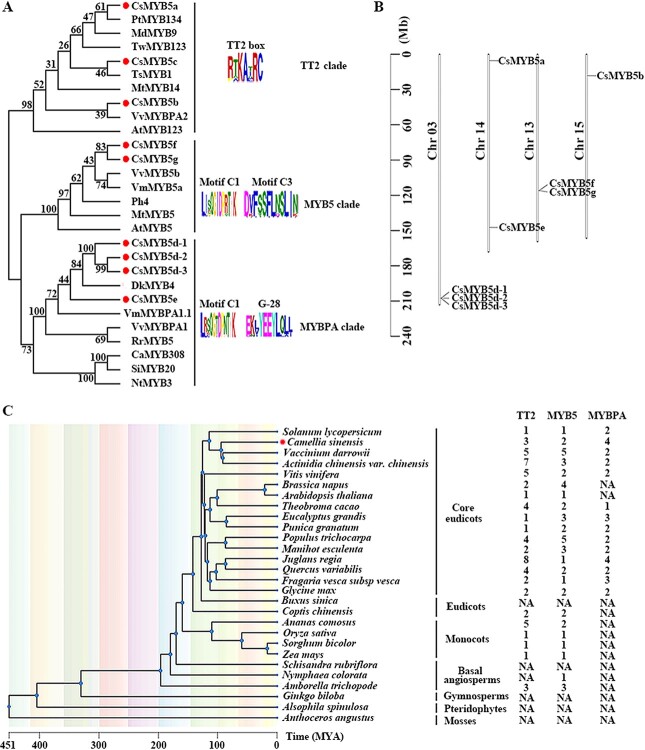
Phylogenetic analysis of subgroup 5 R2R3-MYBs in tea plant. (A) Phylogenetic tree and special motifs of subgroup 5 R2R3-MYB transcription factors from different plant species. The phylogenetic tree was generated using the neighbor-joining method with the MEGA-X version 8 program. The numbers indicate bootstrap values for 1000 replicates. GenBank accession numbers of the MYB proteins are as follows: clade I: CsMYB5a (KAF5936438.1), MdMYB9 (ABB84757), PtMYB134 (ACR83705), TwMYB123 (XP_038712692.1), CsMYB5b (XP_028089226.1), CsMYB5c (XP_028121456.1), TsMYB1 (XP_043705230.1), MtMYB14 (XP_013458423), VvMYBPA2 (ACK56131), AtMYB123 (Q2FJA2); clade II: CsMYB5f (XP_028074710.1), CsMYB5g (XP_028074711.1), VvMYB5b (NP_001267854.1), VmMYB5a (MT316029), PH4 (AAY51377), MtMYB5 (XP_003601609.3), AtMYB5 (NP_187963.1); clade III: VvMYBPA1 (CAJ90831), RrMYB5 (AYP10274), PtMYB115 (XP_002302644), VmMYBPA1.1 (QWW89542), CsMYB5d-1 (GWHPASIV011134), CsMYB5d-2 (GWHPASIV011135), CsMYB5d-3 (GWHPASIV011136), DkMYB4 (AEC11088.1), CsMYB5e (XP_028116920.1) CaMYB308 (XP_016552469), SiMYB20 (XP_004244728.1) NtMYB3 (XP_016440837.1). (B) Chromosome location of CsMYB5s. Chromosome information on CsMYB5s was obtained from the genome of Tieguanyin and chromosome localization was performed by TBtools. (C) Distribution of three types of MYB5 in different species. Plant phylogeny was performed using the online software TIMETREE 5 and all chromosome information on the MYB5 family was obtained from NCBI and is shown in Supplementary Data Table S3.

To fully identify the members of *CsMYB5*s in tea plants, firstly the hidden Markov model (HMM) was used for screening all MYB transcription factors in the genome of *Tieguanyin* based on the model of PF00249 in NCBI [National Center for Biotechnology Information (nih.gov)]. A total of 166 MYB transcription factors were identified (Supplementary Data Table S1). Secondly, the subgroup 5 R2R3-MYBs from seven species were used to identify the CsMYB5 family based on the protein BLAST (Supplementary Data Table S2). The results showed that there were at least nine genes belonging to subgroup 5 R2R3-MYBs in the tea plants, designated *CsMYB5a*, *CsMYB5b*, *CsMYB5c*, *CsMYB5d-1*, *CsMYB5d-2*, *CsMYB5d-3*, *CsMYB5e*, *CsMYB5f*, and *CsMYB5g.* In order to classify the CsMYB5 family, phylogenetic tree analysis was conducted. The result showed that the CsMYB5 family could be divided into three subgroups, namely TT2 type, MYB5 type, and MYBPA type (Fig. 1A). Amino acid alignments indicated that all CsMYB5s contained R2R3 imperfect repeats responsible for target DNA binding and the conserved motif [D/E]Lx2[R/K]x3Lx6Lx3R at the N-terminal regions, which is required for interaction with bHLH co-factors (Supplementary Data Fig. S1). By analyzing the three types of conservative motifs of MYB5 through the online software MEME, we found that the TT2-type MYBs contain a TT2-box ([R/P]xKAxRC); MYB5-type MYBs contain the motifs C1 (Lx2QG[I/T]DPxTHK) and a special motif, C3 ([N/D]D[V/K]F[S/T]SFL[N/D]SLI[N/K]); and the MYBPA-type MYBs include the motif C1 and a distinct conserved G-28 motif (L[E/DKLYEEYL[Q/E][L/V]L]). The motif C1 (Lx2QG[I/T]DPxTHK), also known as the GIDP motif, has been reported not only in subgroup 5 R2R3-MYBs, but also in subgroup 4 R2R3-MYBs [23]. These motifs in the C-terminal region are listed in Fig. 1A.

To determine whether gene expansion of subgroup 5 R2R3-MYBs exists in tea plants, a comparative analysis among different species was conducted. We obtained the whole-genome sequences of subgroup 5 R2R3-MYB members from the genome databases of 28 species. The 28 plants were distributed in mosses, pteridophytes, gymnosperms, basal angiosperms, monocots, eudicots, and core eudicots and the chromosome location information of MYB5s family genes of the above plants is displayed in Supplementary Data Table S3. The results showed that the subgroup 5 R2R3-MYBs of eudicots, such as *A. thaliana*, *Brassica napus*; monocots, such as *Oryza sativa*, *Sorghum bicolor*, *Musa nana* Lour., and *Ananas comosus*; and basal angiosperms, such as *Amborella trichopoda*, had only two subgroups, including TT2 type and MYB5 type. However, in tannin-rich dicots, such as *V. vinifera*, *Vaccinium darrowii*, *Punica granatum*, *Eucalyptus grandis*, *Juglans regia*, *Populus trichocarpa*, and *C. sinensis*, the members of subgroup 5 R2R3-MYBs were significantly expanded in number, with an additional MYBPA type (Fig. 1C). In other words, the MYBPA clade exists mainly in tannin-rich eudicots but not in monocots or older basal angiosperms. Interestingly, we did not find homologous genes of the MYB5 family in some ancient species, such as gymnosperms, pteridophytes, and mosses. The plant MYB5 probably first appeared in basal angiosperms. In tannin-rich plants, gene expansion of the TT2 type in *Actinidia chinensis* (seven) and *J. regia* (eight) is significant. Compared with other tannin-rich dicots plants, the MYBPA type of tea plants showed gene expansion, especially *CsMYB5d*, belonging to the MYBPA type, which had three copies (*CsMYB5d1–3*) that shared 98.86% identity and were oriented in the same direction on chromosome 3 to form a gene cluster (Fig. 1B).

*CsMYB5c* has not been cloned and *CsMYB5d* has three copies (three transcripts and promoters) in the genome, which increases the difficulty of functional verification of *CsMYB5d*. Therefore, we identified nine members of the MYB5 family in the genome of tea plant and carried out functional verification of *CsMYB5a/b/e/f/g.*

### CsMYB5s are nucleus-localized transcriptional activators

**Figure 2:**
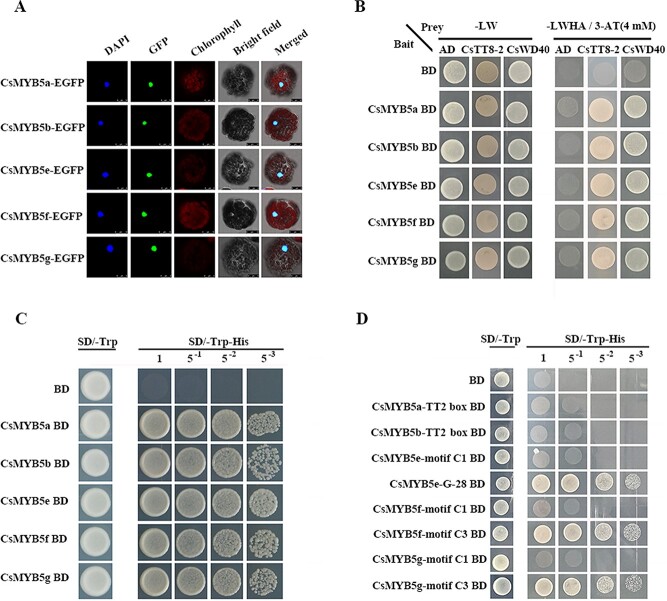
Transcriptional activation region and subcellular localization and protein interaction of CsMYB5s. (A) Subcellular localization of CsMYB5s using protoplasts of *Arabidopsis*. *CsMYB5s-EGFP* fusion gene was transiently expressed in *Arabidopsis* protoplasts. DAPI, nuclear marker; GFP, GFP fluorescence; Chlorophyll, chlorophyll autofluorescence; Bright field, a complete protoplast cell; Merge, combined fluorescence from GFP, chlorophyll, and bright fields. Scale bars = 10 μm. (B) Protein interaction of CsMYB5s with CsTT8-2 and CsWD40 verified through yeast two-hybrid assays. CsMYB5s were fused to the DNA-binding domain (BD), and CsTT8-2 and CsWD40 were fused to the GAL4 activation domain (AD) to test the protein interaction of CsMYB5s with CsTT8-2 and CsWD40. AH109 yeast strains were co-transformed with empty vector pGBKT7 or pGADT7 as negative control. 3-AT, 3-amino-1,2,4-triazole. (C) Transactivation activity of CsMYB5 members. Full-length protein of CsMYB5s fused to the DNA-binding domain was transformed into yeast cells (strain AH109) and grown on SD/−Trp−His. (D) Transcriptional activation capacity of TT2-box, motif C1, motif C3, and G-28 in three types of CsMYB5s.

To investigate the subcellular localization of *CsMYB5a/b/e/f/g*, the opening reading frame (ORF) of EGFP was fused to the C-terminus of CsMYB5s and introduced into the protoplast of *Arabidopsis*. The results showed that the CsMYB5-EGFP fusion proteins were localized in the nucleus (Fig. 2A). In addition, the yeast two-hybrid assay indicated that all five CsMYB5s could interact with CsTT8-2 (basic helix–loop–helix, bHLH) and CsWD40 to form the MYB-bHLH-WD40 complex (MBW) to regulate the expression of downstream target genes (Fig. 2B).

To determine whether CsMYB5s proteins act as transcriptional activators, the ORFs of CsMYB5s from three types, including *CsMYB5a*, *CsMYB5b*, *CsMYB5e*, *CsMYB5f*, and *CsMYB5g*, were fused to the DNA-binding domain (BD) and then transformed into yeast cells (strain AH109). The results showed that the transformants containing pBD-CsMYB5s under series dilution grew well in SD medium lacking tryptophan and adenine, while the growth of control yeast cells containing only pBD was inhibited (Fig. 2C). This result shows that all five CsMYB5s have transactivational activity in yeast. To determine whether the conserved motifs at the C-terminal regions of CsMYB5s have transcriptional activation capability, several 25-amino acid gene fragments containing the TT2-box and the C1, C3, and G-28 conserved motifs were cloned for further experiments. Transactivational analysis showed that conserved motifs G-28 in CsMYB5e protein and motif C3 in CsMYB5f and CsMYB5g protein had transactivational activity, but TT2-box in CsMYB5a and CsMYB5b protein and motif C1 in CsMYB5e, CsMYB5f, and CsMYB5g protein showed no transactivational activity (Fig. 2D).

The above experiments show that *CsMYB5a/b/e/f/g* from tea plants are nucleus-localized transcriptional activators that function through the MBW complex.

### Diversity of CsMYB5 gene expression patterns in tea plants

Monomeric catechins mainly accumulate in the young leaves of tea plants and are rarely distributed in the roots of tea plants, whereas polymerized catechins or PAs are the opposite [24]. Both monomeric C and polymerized C accumulation are associated with the transcript level of subgroup 5 R2R3-MYBs in many species [11, 12].

To better understand the tissue-specific gene expression of *CsMYB5a/b/e/f/g*, the expression levels of CsMYB5s in the leaves and roots were analyzed by qRT–PCR (Supplementary Data Fig. S2). At the same time, GUS staining analysis was used to detect CsMYB5 promoter activity in the aerial parts and roots of transgenic *Arabidopsis* seedlings (Supplementary Data Fig. S2). The results of qRT–PCR showed that the TT2-type CsMYB5s, including *CsMYB5a* and *CsMYB5b*, had a similar expression pattern, which showed a decreasing trend with leaf development. *CsMYB5e* was mainly expressed in the roots. In contrast, *CsMYB5f* and *CsMYB5g* were expressed at higher levels in the mature and old leaves. GUS staining revealed that all *CsMYB5*s were expressed in the aerial parts, but *CsMYB5e* showed the highest intensity of GUS staining in roots.

**Figure 3:**
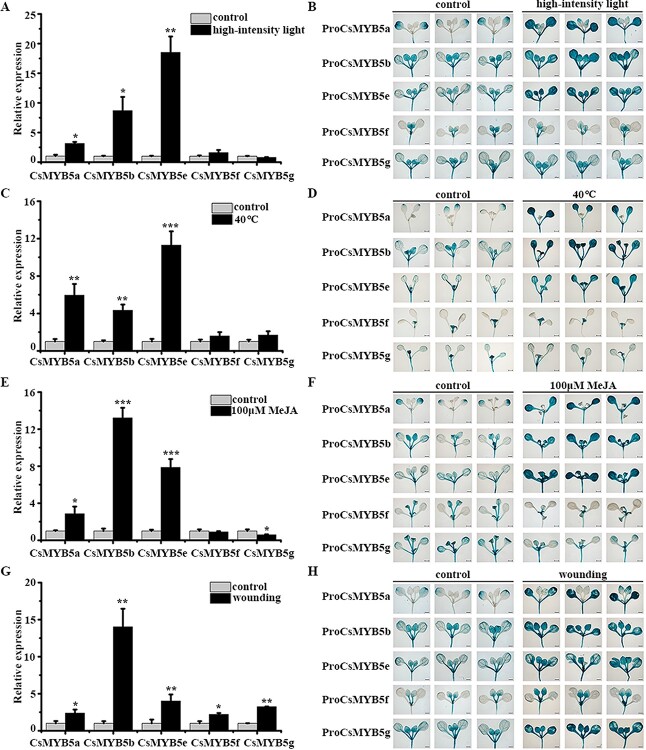
Three types of CsMYB5s in tea plants responded to different stress treatments. (A, C, E, and G) qRT–PCR analysis of gene expression of five CsMYB5s in control, high-intensity light, high temperature, 100 μM MeJA, and wounding. The control groups were treated under fluorescent lamps with a 14-h light/10-h dark photoperiod at 24°C. All samples were collected for gene expression analysis after 48 h. Means were calculated from three repeats and error bars reflect standard deviations. Asterisks indicate significant differences using Student’s *t*-test (*n* = 3, ^*^P < .05, ^**^P < .01,^ ***^P < .001). (B, D, F, and H) Histochemical analysis of β-glucuronidase (GUS) activity of the promoters of CsMYB5s after high-intensity light, high temperature, 100 μM MeJA, and mechanical wounding treatments. All control *Arabidopsis* seedlings were cultured in an environment with a 14-h light/10-h dark photoperiod at 10 000 lux and 22°C. All *Arabidopsis* seedlings were collected for GUS staining after 48 h of treatment. Bars = 2 mm.

To understand the differential expression of *CsMYB5a/b/e/f/g* under biotic and abiotic stresses, we examined the gene expression and promoter activity of CsMYB5s under various stresses, such as high-intensity lights, high temperatures, mechanical damage, fungal infections, low temperatures, salts, droughts, MeJA, and MeSA. qPCR analysis revealed that treatment with high-intensity light, high temperature, mechanical wounding, and MeJA significantly upregulated the expression of *CsMYB5a*, *CsMYB5b*, and *CsMYB5e*, whereas the expression of *CsMYB5f* and *CsMYB5g* was only induced by wounding treatment (Fig. 3A, C, E, and G). The results of the promoter viability analysis of CsMYB5s were consistent with those of qRT–PCR in tea plants under these treatments (Fig. 3B, D, F, and H).

The other treatments, including low temperature (4°C), 150 mM NaCl, 200 mM mannitol, 5 μM ABA, and 100 μM MeSA, did not affect the GUS staining of transgenic *A. thaliana* seedlings with CsMYB5 promoters compared with the control (Supplementary Data Fig. S3).

The above results demonstrate the diversity of *CsMYB5a/b/e/f/g* gene expression patterns in tea plants. In particular, *CsMYB5a/5b*/*5e*, which are highly expressed in leaves and roots, are induced by high temperature, high-intensity light, MeJA, and wounding treatment, while *CsMYB5f/5 g* expressed in mature leaves was only induced by mechanical wounding.

### Identification of target genes regulated by CsMYB5s in tea plants using transient overexpression and antisense oligonucleotide interference

**Figure 4:**
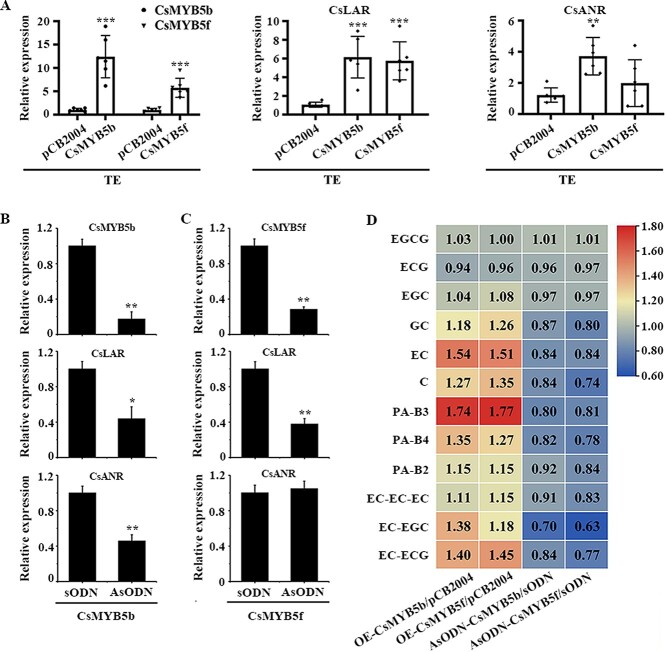
Transient overexpression and AsODN interference of *CsMYB5b* and *CsMYB5f* affected PA biosynthesis in tea plants. (A) qRT–PCR analysis of mRNA level of *CsMYB5b*, *CsMYB5f*, *CsLAR*, and *CsANR* in tea plant leaves that transiently overexpressed *CsMYB5b* and *CsMYB5f*. (B, C) Gene expression of *CsMYB5b*, *CsMYB5f*, *CsLAR*, and *CsANR* in tea plant leaves treated with AsODN interference of *CsMYB5b* (B) and *CsMYB5f* (C). (D) The heat map represents the ratio of the contents of C and PA in tea leaves that overexpressed or interfered with *CsMYB5b* or *CsMYB5f* to that of controls. PA-B2, PA-B3, PA-B4, EC-ECG, EC-EGC, and EC-EC-EC are dimeric or trimeric C monomers. PA-B2 (epicatechin-epicatechin), PA-B3 (catechin-epicatechin), PA-B4 (catechin-catechin), EC-ECG (epicatechin-epicatechin gallate), EC-EGC (epicatechin-epigallocatechin), EC-EC-EC (epicatechin-epicatechin-epicatechin). Means were calculated from six repeats and error bars reflect standard deviations. Asterisks indicate significant differences using Student’s *t*-test (*n* = 6, ^*^P < .05, ^**^P < .01, ^***^P < .001).

In order to verify the effects of CsMYB5s on PA-related genes and metabolism, we transiently overexpressed *CsMYB5a/b/e/f/g* in tea plant leaves and further detected the gene expression of CsMYB5s and their target genes, including *CsLAR* and *CsANR*. Unexpectedly, *CsMYB5a/b/e* could significantly improve transcription levels of *CsLAR* and *CsANR*, but *CsMYB5f* and *CsMYB5g* only significantly increased gene expression levels of *CsLAR* and did not affect gene expression of *CsANR* in their transgenic tea leaves compared with controls (Supplementary Data Fig. S4). In tea plant, antisense oligonucleotide (AsODN) experiments have been widely used to verify the biological function of certain genes by interfering with gene expression of responding genes [25]. AsODN experiments with *CsMYB5a/b/e/f/g* were also conducted in tea plant leaves to further verify the regulation by CsMYB5s of the expression of *CsLAR* and *CsANR* genes. However, there was no successful interference with *CsMYB5a/e/g*, so we analyzed gene expression and flavonoid metabolism in tea leaves with transient overexpression and antisense interference of *CsMYB5b* and *CsMYB5f*. The results showed that *CsMYB5b* and *CsMYB5f* were successfully overexpressed in tea plant leaves, and their gene expression increased by 12.23- and 6.08-fold, respectively, compared with the control. In tea leaves with transient overexpression of *CsMYB5b* and *CsMYB5f*, the gene expression of *CsLAR* was increased by 6.17- and 6.08-fold, respectively. Unexpectedly, the gene expression of *CsANR* was significantly upregulated in *CsMYB5b* transient overexpression tea leaves, while there was no significant difference in *CsMYB5f* transient overexpression tea leaves, compared with the control (Fig. 4A). The result of antisense interference of *CsMYB5b* and *CsMYB5f* showed that the gene expression levels of *CsMYB5b* and *CsMYB5f* were significantly suppressed in tea leaves treated with AsODN-CsMYB5b and AsODN-CsMYB5f compared with the tea leaves treated with sense oligonucleotides of CsMYB5b (sODN-CsMYB5b) and CsMYB5f (sODN-CsMYB5f) (Fig. 4B and C). As expected, *CsLAR* and *CsANR* were significantly decreased in AsODN-CsMYB5b tea leaves, while *CsLAR* was obviously decreased and *CsANR* showed no significant difference in AsODN-CsMYB5f tea leaves, compared with the control (Fig. 4B and C).

In summary, the transient overexpression of *CsMYB5a/b/e/f/g* showed that *CsMYB5a/b/e* could significantly promote the gene expression of *CsLAR* and *CsANR*, while *CsMYB5f* and *CsMYB5g* could only significantly promote the gene expression of *CsLAR* rather than *CsANR* in tea plant. The gene expression levels of *CsLAR* and *CsANR* in AsODN-CsMYB5b and AsODN-CsMYB5f tea leaves were the opposite of those in *CsMYB5b* and *CsMYB5f* transiently overexpressing tea leaves. These results suggest that different members of the CsMYB5 family regulate the target gene *CsANR* differently.

UPLC–MS/MS analysis showed that *CsMYB5b* and *CsMYB5f* did not affect the contents of gallacylated catechins EGCG and ECG, but increased the contents of non-gallic catechins EC, C, GC, and polymerized C PA (Fig. 4D). These results suggest that *CSMYB5b* and *CsMYB5f* mainly increase the content of non-gallic catechins and PAs rather than gallacylated catechins.

### *CsMYB5a/b/e* directly activated the promoters of *CsLAR* and *CsAN*R whereas *CsMYB5f/g* only activated the promoter of *CsLAR*

**Figure 5:**
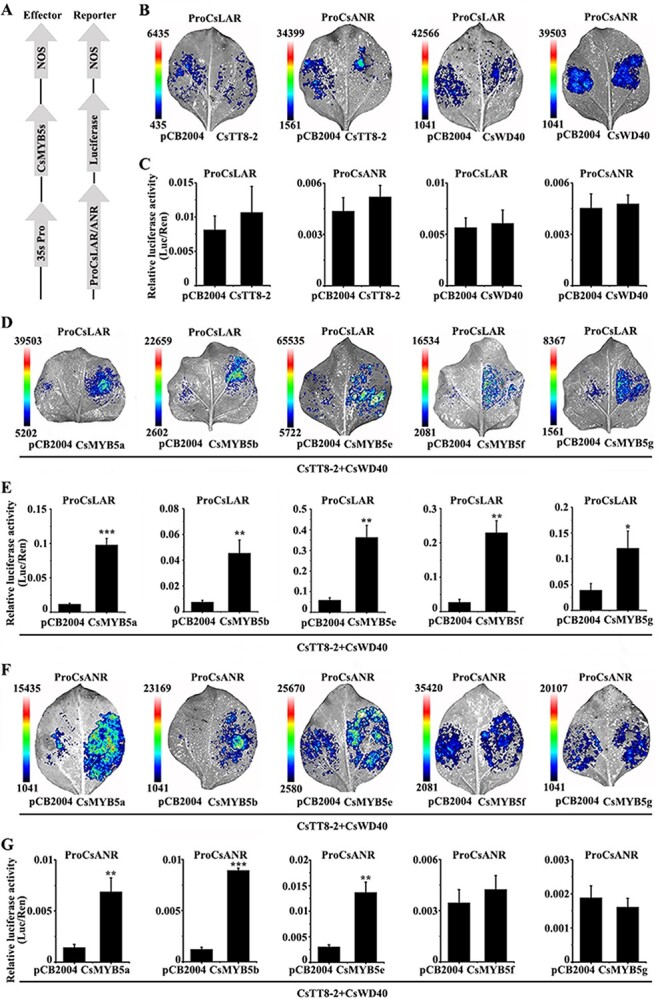
CsMYB5s activate the promoters of *CsLAR* and *CsANR* in the dual-luciferase assay. (A) Schematic representation of the CsMYB5s and the promoter constructs used for the dual-luciferase assay. (B, D, and F) Representativ*e in vivo* imaging of the promoters of *CsLAR* and *CsANR* activated by CsMYB5s together with *CsTT8-2* and *CsWD40* (D and F) or *CsTT8-2* (B) or *CsWD40* (B). (C, E, and G) Dual-luciferase assay results and LUC/REN ratios are shown. *Renilla* luciferase was used to normalize luciferase activity. Means were calculated from three repeats and error bars reflect standard deviations. Asterisks indicate significant differences according to *t*-tests at three significance levels (*n* = 3, ^*^P < .05, ^**^P < .01, ^***^P < .001).

AtTT2 interacts with AtTT8 and AtWD40 to form a protein complex, which can significantly improve the activation of promoters of *AtANR* [26]. *AtTT8* has two homologous genes in tea plant, named *CsTT8* and *CsTT8-2*; *CsTT8* did not promote PA accumulation in the seeds of *tt8* mutants, whereas *CsTT8-2* did (Supplementary Data Fig. S5). Moreover, *CsWD40* has been reported to promote anthocyanin and PA biosynthesis in transgenic tobacco and *A. thaliana* [27]*.* To verify the target genes of CsMYB5s in tea plants, we cloned the promoters of *CsLAR* and *CsANR* and constructed them into the pGreenII 0800 vector for the dual-luciferase reporter assay. The results showed that *CsTT8-2* or *CsWD40* alone could not activate the promoters of *CsLAR* and *CsANR* (Fig. 5B and C). However, the promoter activity of *CsLAR* was significantly upregulated ~8.32-, 6.04-, 6.18-, 8.51-, and 3.08-fold by *CsMYB5a*, *CsMYB5b*, *CsMYB5e*, *CsMYB5f*, *CsMYB5g*, respectively, co-expressed with *CsTT8-2*, and *WD40* (Fig. 5D and E). Unsurprisingly, the promoter activity of *CsANR* was significantly upregulated ~4.81-, 7.34-, and 4.53-fold by *CsMYB5a*, *CsMYB5b*, and *CsMYB5e* but not *CsMYB5f* and *CsMYB5g* in the presence of *CsTT8-2* and *CsWD40* (Fig. 5F and G). To summarize, *CsMYB5a/b/e/f/g* can activate the promoter of *CsLAR* but only *CsMYB5a/b/e* can activate the promoter of *CsANR* but *CsMYB5f* and *CsMYB5g* cannot. These results suggest that the CsMYB5 family has functional differences in regulating the promoters of PA-related genes, especially the promoter of *CsANR.*

### Verifying the subtle differences in function of CsMYB5s via the tobacco genetic transformation system

**Figure 6:**
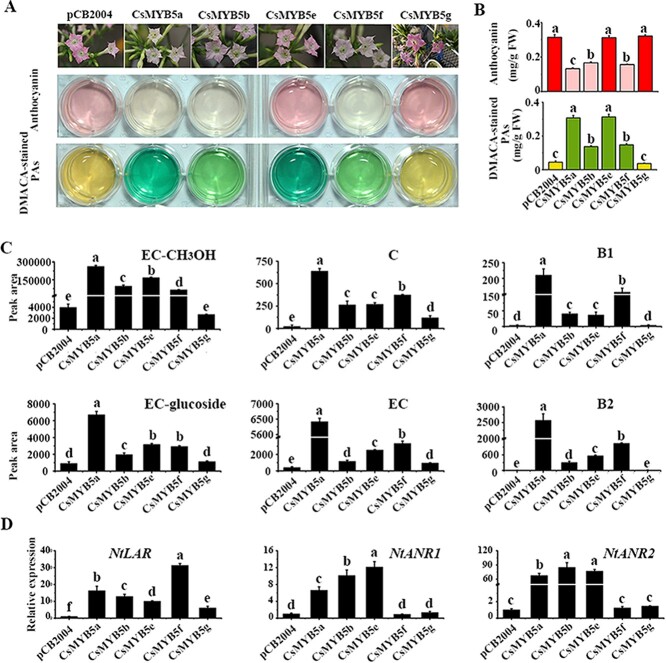
Differential accumulation of phenolic compounds in three types of CsMYB5-overexpressing transgenic tobacco. (A) Phenotypes of empty vector control (pCB2004) and CsMYB5s transgenic tobacco flowers (top row), coloration of anthocyanins (second row), and DMACA-stained PA (third row). (B) Contents of anthocyanin and DMACA-stained PA at 530 and 640 nm, respectively. (C) Related content of EC-CH_3_OH (*m*/*z* 319), C (*m*/*z* 289), PA B1 (*m*/*z* 577), EC-Glu (*m*/*z* 451), EC (*m*/*z* 289), and PA B2 (*m*/*z* 577) in control and CsMYB5-overexpressing tobacco flowers analyzed through MS-based multiple reaction monitoring. (D) Relative expression of flavonoid biosynthetic pathway genes in CsMYB5-overexpressing tobacco flowers through qRT–PCR analysis. LAR, leucoanthocyanidin reductase; ANR, anthocyanidin reductase. All data are means of three biological replicates and error bars denote standard deviation. Different letters indicate significant differences among groups at α = 0.05 as determined by Duncan’s multiple range test (*n* = 3). FW, fresh weight.

Our previous study indicated that both *CsMYB5a* and *CsMYB5e* significantly increased dimethylaminocinnamaldehyde (DMACA)-stained PAs in transgenic tobaccos [8]. To compare the functional differences of the five CsMYB5s in PA accumulation, they were overexpressed in tobacco under the control of the 35S promoter. Compared with the flowers of transgenic tobacco transformed with the empty vector pCB2004 (negative control), *CsMYB5a/5b/5f*-overexpressing tobacco displayed pale red flowers, whereas *CsMYB5e/5g* did not affect flower color (Fig. 6A). Correspondingly, anthocyanin content was significantly decreased in *CsMYB5a/5b/5f* transgenic tobacco plants (Fig. 6B). In contrast, DMACA-stained PA levels were strongly increased in *CsMYB5a/5b/5e/5f*-overexpressing tobacco flowers, reaching 0.307, 0.267, 0.313, and 0.148 mg g^−1^ fresh weight, respectively, whereas CsMYB5g did not differ in DMACA-stained PA accumulation compared with the control (Fig. 6A and B).

DMACA-stained PA includes EC, C, epicatechin carbocation (EC-CH_3_OH), PA B1 (dimer of C and EC-CH_3_OH), and PA B2 (dimer of EC and EC-CH_3_OH) [2]. UPLC–MS/MS was used to further identify which component accumulated in *CsMYB5*s-overexpressing tobacco flowers. The results indicated that EC and C, EC-CH_3_OH, and their dimers, proanthocyanin B1 and B2, were highly accumulated in *CsMYB5a/5b/5e/5f* transgenic tobacco flowers compared with the control (Fig. 6C). However, only small amounts of EC and C were detectable in *CsMYB5g* transgenic tobacco. Taken together, these results indicated that *CsMYB5a/5b/5e/5f* dramatically upregulated PA biosynthesis, while the function of *CsMYB5g* in PA biosynthesis was less significant than that of other CsMYB5s.

Our previous study showed that *CsLAR* could promote the accumulation of EC and C in transgenic tobacco, and *CsANR* catalyzed the formation of EC-CH_3_OH involved in PA biosynthesis [2]. To confirm the target genes of *CsMYB5*s, qRT–PCR analysis was performed to analyze the expression levels of key genes involved in PA biosynthesis (Fig. 6D). The results showed that *NtLAR* expression was upregulated in all transgenic plants, whereas *NtANR1* and *NtANR2* expressions were upregulated only in *CsMYB5a/5b/5e* transgenic tobacco. In particular, *NtANR2* was dramatically upregulated ~67.6-, 84.8-, and 76.8-fold in *CsMYB5a/5b/5e* transgenic tobacco, respectively. *NtLAR* was upregulated ~16.3-, 12.8-, 10-, 31.2-, and 6-fold in *CsMYB5a/5b/5e−/5f/5g* transgenic tobacco, respectively*.* The expression levels of other genes in the flavonoid pathway, such as *chalcone synthase* (*CHS*), *chalcone isomerase* (*CHI*), *flavanone 3-hydroxylase* (*F3H*), *flavonoid 3′-hydroxylase* (*F3′H*), *flavonoid 3′,5′-hydroxylase* (*F3′5′H*), *dihydroflavonol-4-reductase* (*DFR*), *flavonol synthase* (*FLS*), and *anthocyanidin synthase* (*ANS*), were less affected than those of *NtLAR* and *NtANR* (Supplementary Data Fig. S6).

In summary, *CsMYB5a/5b/5e/5f* significantly upregulated PA biosynthesis, while *CsMYB5g* slightly upregulated PA accumulation in transgenic tobacco. Another significant difference was that CsMYB5a/5b/5f significantly reduced anthocyanin content in flowers of transgenic plants, while CsMYB5e/5g did not affect anthocyanin content. In tobacco flowers overexpressing CsMYB5f/5g, the expression of *NtANR* was not upregulated compared with the control, whereas *CsMYB5g* slightly induced the expression of *NtLAR*.

### R2R3 domains of CsMYB5s determine their subtle functional differences in activating *ANR*

The transparent testa 2 gene (*TT2*) promotes PA biosynthesis by positively regulating the expression of *ANR* (*BANYULS*, *BAN*) in *A. thaliana* and is responsible for the brown pigment in the seed coat [15]. Therefore, the *tt2* mutant appears with yellow seeds because of the absence of brown pigment in the seed coat. To further verify the functional differences between CsMYB5s in PA biosynthesis, a genetic complementation experiment was performed by overexpressing CsMYB5s in the *tt2* mutant. Results showed that both TT2-type MYB5 (*CsMYB5a*, *CsMYB5b*) and MYBPA-type MYB5 (*CsMYB5e*) promoted DMACA-stained PA accumulation (Supplementary Data Fig. S7). However, *tt2* mutants overexpressing *CsMYB5f* and *CsMYB5g* did not complement the PA-deficient phenotype (Supplementary Data Fig. S7).

Furthermore, the transcript level of *AtANR* (*BAN*) in all CsMYB5-transgenic *tt2* mutants was determined by qRT–PCR (Supplementary Data Fig. S8). As expected, overexpression of *CsMYB5a*, *CsMYB5b*, and *CsMYB5e* in the *tt2* mutant led to the upregulated expression of *AtANR* in transgenic plants, but not in *tt2* mutants overexpressing *CsMYP5f* and *CsMYB5g* (Supplementary Data Fig. S8). This result was consistent with that of CsMYB5-transgenic tobacco and transiently overexpressing tea leaves.

**Figure 7:**
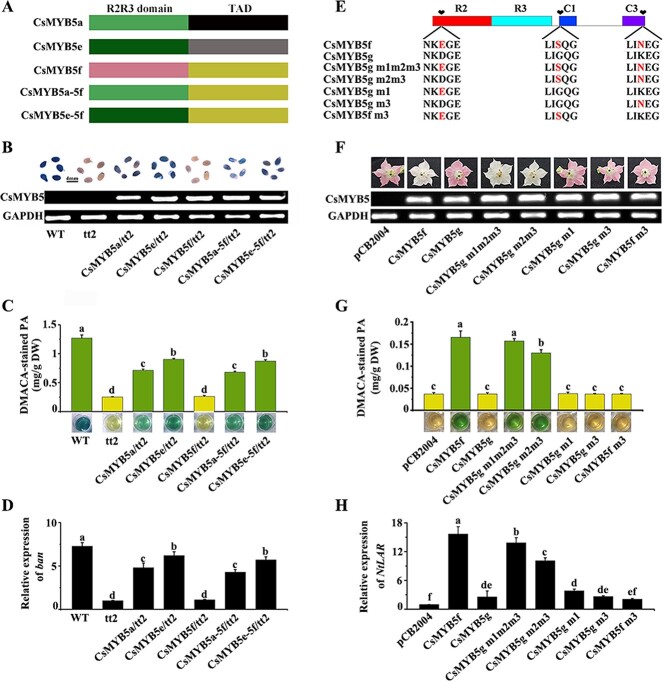
The R2R3 domain and conservative motifs in the C-terminus of CsMYB5s determine the functional diversity of the MYB5 family. (A) Schematic overview of chimeric constructs generated to exchange the R2R3 domain between CsMYB5a, CsMYB5e, and CsMYB5f. (B) DMACA staining in mature seeds of *A. thaliana*. (C, G) Contents of DMACA-stained PA at 640 nm. (D) Relative gene expression of *BAN* through qRT–PCR analysis. (E) Partial alignment of CsMYB5f, CsMYB5g, and their respective point mutants. Mutated residues of m1, m2, and m3 are marked. (F) Phenotypes of tobacco flower overexpressing *CsMYB5f*, *CsMYB5g* and they point mutation. (H) Relative expression of *NtLAR* in transgenic tobacco flowers through qRT–PCR. All data are means of three biological replicates, and error bars denote the standard deviation. Different letters mean the significance level at *P* < .05 based on Tukey’s honestly significant difference test (*n* = 3). DW, dry weight; FW, fresh weight.

The R2R3 domain is responsible for binding to the promoter of the target gene [28]. To explore why *CsMYB5f* and *CsMYB5g* could not activate *ANR* in tobacco or *A. thaliana*, several R2R3 domain-swapping experiments were conducted on CsMYB5f. The N-terminus containing the R2R3 domain of CsMYB5f was replaced with the N-terminus of CsMYB5a and CsMYB5e to generate CsMYB5a-5f and CsMYB5e-5f constructs, respectively (Fig. 7A). *CsMYB5a-5f* and *CsMYB5e-5f* constructs were then overexpressed in the *tt2* mutant under the control of the 35S promoter. The results showed that overexpression of both *CsMYB5a-5f* and *CsMYB5e-5f* in the *tt2* mutant produced the same phenotype as *CsMYB5a* and *CsMYB5e*; i.e. the DMACA-stained PA content increased in the seed coat (Fig. 7B and C). The results of qRT–PCR showed that the gene expression levels of *BAN* were upregulated 4.8-, 6.2-, 4.2-, and 5.7-fold in *CsMYB5a*-, *CsMYB5e-*, *CsMYB5a-5f-*, and *CsMYB5e-5f-*overexpressing *tt2* mutants, respectively, compared with that of the *tt2* mutant (Fig. 7D).

Taken together, the results of the R2R3 substitution experiment indicated that the difference in the R2R3 domain of the three types of CsMYB5s affected their ability to regulate the gene expression of *ANR* and finally determined their functional differences in PA biosynthesis.

### Key amino acids of CsMYB5f and CsMYB5g determine their subtle differences of function in promoting PA biosynthesis

It has been reported that the conserved domain in the C-terminus of R2R3-MYBs is critical to their function [29, 30]. *CsMYB5f* and *CsMYB5g* belong to the MYB5-type clade and have highly similar, conserved domains. However, *CsMYB5g* had a weaker effect on the regulation of PA synthesis than *CsMYB5f*. Hence, to further explore the functional differences between *CsMYB5f* and *CsMYB5g* in PA biosynthesis, a series of site-directed mutations on conserved C1 and C3 domains in the C-terminus and R2 domain in the N-terminus were carried out (Fig. 7E). Three potentially functional amino acids located in these conserved domains were selected for further functional verification. They were named m1 (D46 of CsMYB5g and E53 of CsMYB5f), m2 (G132 of CsMYB5g and S138 of CsMYB5f), and m3 (N278 of CsMYB5g and K281 of CsMYB5f). The mutated *CsMYB5g* and *CsMYB5f* were then overexpressed in tobacco under the control of the 35S promoter.

The results showed that both *CsMYB5g m1m2m3*- and *CsMYB5g m2m3*-overexpressing tobacco plants produced white flowers and accumulated a lot of DMACA-stained PA, whereas *CsMYB5g m1*- and *CsMYB5g m3*-overexpressing tobacco plants exhibited no difference compared with *CsMYB5g* (Fig. 7F and G). Quantitative analysis with UPLC–MS/MS showed that the contents of EC, C, EC-CH_3_OH, and proanthocyanin B1 and B2 in *CsMYB5g m1m2m3*-overexpressing transgenic tobacco flowers increased significantly, which was almost equal to their contents in *CsMYB5f*-overexpressing transgenic tobacco flowers (Supplementary Data Fig. S9). Furthermore, qRT–PCR analysis revealed that expression levels of *NtLAR* increased 15.6-, 13.8-, and 10.1-fold in *CsMYB5f*-, *CsMYB5g m1m2m3*-, and *CsMYB5g m2m3*-overexpressing transgenic tobacco, respectively (Fig. 7H). These results indicate that G132 and K278 in CsMYB5g affect PA biosynthesis and the expression of *NtLAR* in tobacco. Conversely, *CsMYB5f m3* transgenic tobacco could not produce DMACA-stained PA or upregulate the expression of *NtLAR* (Fig. 7G and H). This indicates that N278 is essential for the function of CsMYB5f in PA biosynthesis in tobacco. As expected, the gene expression of *NtANR1* and *NtANR2* was not significantly upregulated in transgenic tobacco with all CsMYB5f and CsMYB5g point mutations, compared with the control (Supplementary Data Fig. S10).

## Discussion

### Evolution of three types of subgroup 5 R2R3-MYBs in plants

Phylogenetic analysis can be used not only to trace the evolutionary route of genes but also to predict the function of proteins based on sequence similarity. MYB TFs with the same motif at the C-terminus have similar functions [23]. From an evolutionary perspective, subgroup 5 R2R3-MYBs in plants did not exist in some ancient species, such as gymnosperms, pteridophytes, and mosses, which indicated that the plant MYB5 probably first appeared in basal angiosperms (Fig. 1C). both TT2-type and MYB5-type clades are distributed in eudicots, monocots, and even basal angiosperms, whereas the MYBPA-type clade exists only in dicotyledonous plants, especially those rich in tannins (Supplementary Data Table S3). Tea plants, as typical tannin-rich plants, are high in ester catechins, and the genes responsible for the synthesis and hydrolysis of ester catechins have been identified [31]. Tannin-rich plants include *V. vinifera*, *Manihot esculenta*, *P. granatum*, *E. grandis*, *J. regia*, *Quercus variabilis*, and *D. kaki.* Moreover, both MYBPA-type and MYB5-type members have a conserved motif C1 in the C-terminus (Fig. 1A). By comprehensively considering evolutionary relationships and C-terminal conserved motifs of the three types of CsMYB5s, we speculate that the MYBPA-type clade may have originated from the MYB5-type clade during plant evolution. We found that gene expansion of the TT2-type subgroup 5 R2R3-MYBs occurred in *V. vinifera*, *A. chinensis*, and *J. regia* (Supplementary Data Table S3)*.* The relationship between gene expansion and PA accumulation requires further investigation.

### Functional commonality and specificity of three types of CsMYB5s in tea plants

Undoubtedly, the main function of subgroup 5 R2R3-MYBs is to regulate the biosynthesis of PAs, in particular, the TT2 type and the MYBPA type. Ectopic expression of *CsMYB5a*, *CsMYB5b*, and *CsMYB5e* in tobacco confirmed that the TT2 type and the MYBPA type in tea plants have similar functions in promoting PA synthesis by upregulating *LAR* and *ANR* (Fig. 6A). However, the functions of anthocyanin biosynthesis in TT2-type members in different species are different; for example, in rose, *RrMYB10* accumulated a lot of anthocyanins in *RrMYB10-*overexpressing tobacco flowers [14], whereas MdMYB9 accumulated large amounts of anthocyanins in apple calluses [32]. In contrast, in *M. truncatula*, overexpression of *MtMYB14* in hair roots significantly reduced anthocyanin content compared with the control [12], whereas in *C. sinensis CsMYB5a-* and *CsMYB5b-*overexpressing tobacco flowers significantly reduced the amount of anthocyanins compared with the control (Fig. 6A and B). The MYBPA-type members in different species showed similar functional differences; for example, *RrMYB5* in rose reduced the accumulation of anthocyanins [14], but *CsMYB5e*, belonging to the MYBPA type, did not affect the accumulation of anthocyanins compared with the control (Fig. 6A and B)*.* The gene expression pattern of 12 *Uridine diphosphate glycosyltransferase* (*UGT*) and 16 *glutathione S-transferase* (*GST*) genes were inconsistent with the accumulation of anthocyanins in *CsMYB5a* and *CsMYB5e* transgenic tobacco [8]. Both TT2 type (*MdMYB9*, *MdMYB12*) and MYBPA type (*MdMYBPA1*) could bind to the promoter of *UFGT* and induce the accumulation of anthocyanin [33]. The homologous genes of *Arabidopsis TT19* (*AtGST*) and apple *MdUFGT* were identified in tea plant and named as *CsGST1*, *CsGST2*, *CsGST3*, *CsUGT1*, and *CsUGT2*. By analyzing their promoter *cis*-acting elements, we found a large number of MYB-binding elements, especially MBSI, involved in flavonoid biosynthetic gene regulation (Supplementary Data Fig. Fig. S11). Subgroup 5 R2R3-MYB regulates anthocyanin biosynthesis differently in different species, which may be due to differences in the regulation of *UGT* and *GST*. This aspect deserves further study.

The biological functions of MYB5 type are diverse in different species. *AtMYB5* in *A. thaliana* is mainly responsible for the accumulation of mucilage in the seed coats and epidermal branches of leaves, but plays a minor role in the accumulation of PAs. In *Petunia*, as a homolog of *AtMYB5*, *PH4* mainly regulates vacuolar acidity [18]. In *Freesia hybrida*, overexpression of *FhMYB5* in both *Freesia* protoplasts and tobacco, could only activate *LAR* and failed to promote the expression of *ANR* [34]. In *C. sinensis*, *CsMYB5f* and *CsMYB5g* did not activate *ANR* in either *A. thaliana* or tobacco (Fig. 6D and Supplementary Data Fig. S8), which is similar to *FhMYB5.* The biological functions of MYB5 family members in different species have both commonality and specificity.

### Differences in conserved domains determine the functional diversity of the three types of CsMYB5s

The R2R3 domain determines its ability to bind to the promoter of the target gene [28]. By swapping the R2R3 domain of CsMYB5a and CsMYB5e with CsMYB5f, we proved that the R2R3 domain of the three types of CsMYB5s determined the functional differences in the regulation of *ANR* (Fig. 7D). The R2 and R3 domains contain the same helix-turn-helix structure, and the last helix in R2 and R3 could bind to DNA [35]. Therefore, we speculated that the key amino acids in the R2R3 domain of the three types of CsMYB5 are located in the last helix of the R2R3 domain.

It has been indicated that MYB TFs with the same motif at the C-terminus have similar functions [23]. The function of the C-terminal conserved motif of MYB TF is often related to transcriptional activation capacity, physical interactions, and post-translational modification [29]. According to our experimental results, the functional differences between CsMYB5f and CsMYB5g were also related to the key amino acids located at the C-terminal conserved motif (Fig. 7E and G). Overexpression of *CsMYB5f* in tobacco resulted in the accumulation of larger amounts of EC, C, EC-CH3OH, PA B1, and PA B2 compared with *CsMYB5g* overexpression (Fig. 6C). Point mutation experiments showed that the function of CsMYB5g with two amino acid mutations in motifs C1 (G132S) and C3 (K278N) was almost the same as that of CsMYB5f in PA biosynthesis (Supplementary Data Fig. Fig. S9). The function of CsMYB5g with a mutation in motif C3 (K278N) was not affected; however, CsMYB5f with a mutation in motif C3 (N281K) did not accumulate DMACA-stained PA (Fig. 7). Based on the above results, we speculate that serine (S) in motif C1 and asparagine (N) in motif C3 determine the functional difference between CsMYB5f and CsMYB5g in accumulating DMACA-stained PA.

However, one aspect of these experimental results remains unexplained. Previous research reported that in tobacco plants overexpression of *CsLAR* could induce the synthesis of EC and C, whereas overexpression of *CsANR* could induce EC-CH3OH accumulation. EC or C automatically polymerizes with EC-CH3OH to produce PAs B2 and B1, respectively [2]. We found that although *CsMYB5f* did not promote *ANR* gene expression in tobacco, overexpression of *CsMYB5f* in tobacco resulted in the accumulation of large amounts of EC-CH3OH, PA B1, and PA B2. The reasons for this require further research and exploration.

### Prospect of PA synthesis induced by stress

Subgroup 5 R2R3-MYBs play a redundant role in regulating PA synthesis. Their differential expression patterns may be worthy of further exploration, especially for induced expression. In different species, the MYB5 family responds to diverse stresses such as high-intensity light, JA, and mechanical wounding. High-intensity light as a stress signal is known to induce PA biosynthesis by subgroup 5 R2R3-MYBs, which has been reported in different plants, such as poplar [11], apple [32], and grape [36]. Furthermore, the molecular mechanism by which high-intensity light induces the expression of MYB5 has been reported. In poplar, *PtrBBX23* and *PtrHY5* interact with each other to form a protein complex that binds and activates the promoter of *PtrMYB115* and then modulates the accumulation of PA in response to high-intensity light [37]. In apples, *MdWRKY41* inhibits PA accumulation by downregulating the expression of *MdMYB12*. However, light-induced *MdHY5* inhibited the expression of *MdWRKY41* and promoted PA biosynthesis [38]. The molecular mechanism by which JA promotes PA biosynthesis by upregulating the expression of subgroup 5 R2R3-MYB has been reported in apples. For example, *MdJAZ1* interacting with *MdTRB1* interferes with the formation of a protein complex between *MdTRB1* and *MdMYB9* and thus inhibits the activation of *MdLAR* and *MdANR* [39]. *MdERF1b* is induced by JA and activates the promoter of *MdMYB9*. Simultaneously, JA can relieve the protein interaction between MdJAZ and MdERF1b by degrading the MdJAZ protein [40]. High-intensity light and JA also induced the expression of *CsMYB5a*, *CsMYB5b*, and *CsMYB5e* in tea plants (Fig. 3A, B, E, and F).

Mechanical wounding promotes PA biosynthesis by inducing gene expression of the MYB5 family, which has been reported in many species. The TT2 type, such as *TT2* in *Arabidopsis* [41], *DkMYB2* in *D. kaki* [42], *PtMYB134* in poplar [19], *MdMYB9* and *MdMYB11* in apple [32], and *RrMYB10* in rose [14], was induced by mechanical wounding to promote PA biosynthesis. The MYBPA type, including *PtMYB115* in poplar [19], *SsMYB3* in coleus [43], and *RrMYB5* in rose [14], also responded to wounding stress and promoted PA biosynthesis by upregulating the expression of *LAR* and *ANR.* In this study, three types of CsMYB5s in tea plants responded significantly to wounding stress, based on the results of qPCR and promoter activity analysis (Fig. 3G and H). We have comprehensively analyzed functional differences and stress-induced expression differences among different members of the CsMYB5 family (Supplementary Data Fig. S12). However, the molecular mechanism by which mechanical wounding induces the expression of subgroup 5 R2R3-MYBs has rarely been reported in plants. Future studies should focus more on the mechanism by which external stimuli regulate the subgroup 5 R2R3-MYBs.

## Materials and methods

### Plant materials and growth

Seedlings of the tea plant cultivar *C. sinensis* ‘Shuchazao’ were cultivated in the Experimental Tea Garden of Anhui Agricultural University (latitude 31.86° N, longitude 117.27° E). Cuttings of tea plant cultivar *C. sinensis* ‘Shuchazao’ were grown in a greenhouse with a 14-h/10-h light/dark photoperiod and a 25/22°C day/night temperature regime for a week, and then a series of treatments were performed; *A. thaliana* Col-0, *tt2* mutant (SALK_005260), and *tt8* mutant (CS111) were used for genetic transformation. They were grown in a greenhouse with a 16-/8-h light/dark photoperiod and a 23/21°C day/night temperature regime. *Nicotiana tabacum* cv. G28 plants were used for genetic transformation and *N. benthamiana* plants were used for the dual-luciferase reporter assay. They were planted in an environmental chamber with a constant 25/22°C day/night temperature regime and 14-/10-h light/dark photoperiod.

### Gene cloning and plant transformation

RNA extraction and cDNA generation were performed following a reported protocol [24]. Based on the cDNA sequences, the ORFs of *CsMYB5a*, *CsMYB5b*, *CsMYB5e*, *CsMYB5f*, *CsMYB5g*, *CsTT8-2*, and *CsWD40* were cloned using the primers listed in Supplementary Data Table S4. These genes were cloned into vector pCB2004 using the Gateway Cloning System (Invitrogen, New York, USA) and then transformed into *Agrobacterium tumefaciens* strain GV3101 using the freeze–thaw method. As previously described, the genetic transformation of *Arabidopsis* and tobacco was conducted using floral-dip and leaf disk protocols, respectively [24].

### Generation of domain-swapping CsMYB5s and site-directed mutagenesis of CsMYB5s

The chimera constructs *CsMYB5a-5f* and *CsMYB5e-5f* were created from *CsMYB5a*, *CsMYB5e*, and *CsMYB5f* in a two-step PCR. Step one involved two rounds of PCR using *CsMYB5a* forward primer and *CsMYB5a-5f* reverse primer to amplify the N-terminal fragment of *CsMYB5a*, then using *CsMYB5a-5f* forward primer and *CsMYB5f* reverse primer to amplify the C-terminal fragment of *CsMYB5f*. Step two involved a single-round PCR with the primers (*CsMYB5a* forward primer and *CsMYB5f* reverse primer) and templates (products of the first-round PCR reactions) to amplify the ORF of *CsMYB5a-5f*. *CsMYB5e-5f* was generated in the same way using different primers.

Site-directed mutagenesis of *CsMYB5f* and *CsMYB5g* was performed as previously described. For double mutation, the single-mutant fragment was used as a template, and for the three-column procedure the double-mutant fragment was used as a template, one mutation at a time. All primers used are listed in Supplementary Data Table S4.

### Quantitative real-time polymerase chain reaction

The qRT–PCR procedure and the calculation method for relative expression have been previously published [24]. The amplification efficiency of the primers was calculated by diluting the concentration of the template in different multiples, and 90–110% amplification efficiency was acceptable. Primers used for qRT–PCR are listed in Supplementary Data Table S4.

### Extraction and detection of anthocyanins and PAs

The following experimental steps were used to extract polyphenols from tea plants, tobacco, and arabidopsis. For tea plant, freeze-dried leaves were ground into powder, then 0.5 g of sample was weighed and 1 ml of 80% methanol was added for ultrasonic extraction for 10 min. After centrifugation at low temperature (10°C) and high speed (12 000 rpm) for 10 min, the supernatant was transferred to a new tube and another 1 ml of 80% methanol was added to the precipitate. After five repetitions, the volume of the extracted sample was fixed to 5 ml. For tobacco and arabidopsis, 0.1 g tobacco flowers or 0.02 g arabidopsis seeds was weighed and 1 ml of 80% methanol plus 0.2% hydrochloric acid (HCl) was added for ultrasonic extraction for 10 min, as mentioned above, to obtain a final volume of 2.0 ml polyphenol extract. The polyphenol extracts of tobacco flowers or *Arabidopsis* seeds were used for detecting the contents of anthocyanins at 530 nm with a spectrophotometer. The PA contents were detected at 640 nm after a DMACA reaction assay. UPLC coupled with triple-quadrupole mass spectrometry (QQQ–MS) (Agilent, CA, USA) was used to quantify monomeric C, EC intermediates, PA dimers (procyanidin B1, B2, B3, and B4), and PA trimers. Extraction and detection of anthocyanins and PAs were based on the previous method [24].

### Histochemical detection of GUS activity

The promoters of CsMYB5s were constructed in the vector pCAMBIA1304 to replace the CaMV35S promoter. The primers used are listed in Supplementary Data Table S4. All *Arabidopsis* seedlings were incubated at 37°C for 12 h, then decolorized with 75% ethanol. The 75% ethanol was changed every 3 h. After three repeats, *Arabidopsis* seedlings were collected and photographed. For each treatment, 12 independent transgenic seedlings were detected; representative observations are presented.

### Dual-luciferase reporter assay

For the dual-luciferase reporter assay, all *CsMYB5*s, *CsTT8-2*, and *CsWD40* were combined into the pCB2004 effector vector under the control of CaMV35S. Promoter fragments of the target gene were cloned into the reporter vector pGreenII 0800. The primers used are listed in Supplementary Data Table S4. All constructs were introduced into the *A. tumefaciens* GV3101 strain. *Agrobacterium tumefaciens* containing effector vectors and reporter vectors was cultured to OD600 = 0.8 and collected by centrifugation at 5000 rpm for 7 min at room temperature. *Agrobacterium tumefaciens* was resuspended and the suspension was centrifuged for collection. After repeating once, the experimental group containing CsMYB5s, CsTT8-2, CsWD40, and the promoter of the target gene, and the control group containing pCB2004 empty vector, CsTT8-2, CsWD40, and the promoter of the target gene were mixed. After 2 h of incubation at room temperature, the mixed *A. tumefaciens* was injected into 5- to 6-week-old *N. benthamiana* leaves. We detected luminescence signals using a CCD imaging apparatus (Lumazone Pylon2048B) after spraying the leaves with luciferin substrate. Meanwhile, we detected the activity of firefly luciferase (LUC) and *Renilla* luciferase (REN) using dual-luciferase assay reagents (Promega, Madison, WI, USA), and the ratio of LUC to REN represented the activity of the promoter.

### **Transactivation analysis in yeas**t

CsMYB5s were examined for the presence of an activation domain by using a yeast-based reporter assay. Full-length CsMYB5 sequences were cloned into the DBD vector, pGBKT7. Subsequently, constructs harboring CsMYB5s were transformed into the AH109 yeast strain. For assessment of transactivational activity, a series of dilutions of the transformed yeast cultures (OD_600_ = 0.6, and then 5^−1^-, 5^−2^-, and 5^−3^-fold serial dilutions) were plated onto SD plates without tryptophan (SD/−Trp) and SD plates without tryptophan and histidine (SD/−Trp−His) for 3 days at 28°C. pGBKT7 empty vector-transformed yeast lacking the ability to activate the GAL1 promoter served as a negative control. The primers used are listed in Supplementary Data Table S4.

### **Subcellular localization of CsMYB5**s

The ORFs of CsMYB5s lacking the stop codon were cloned into the pCAMBIA1305 vector harboring EGFP (enhanced green fluorescent protein). For transient expression analysis, fusion constructs (CsMYB5s-EGFP) were transformed into *Arabidopsis* protoplasts. *Arabidopsis* protoplasts were isolated and transformed, as described by (https://www.ncbi.nlm.nih.gov/pubmed/17585298). EGFP signals were detected using confocal microscopy (Leica DM2000).

### Treatment of tree plant and *Arabidopsis* with the promoters of CsMYB5s

For the mechanical wounding treatment, annual tea cutting under fluorescent lamps with a 14-h light/10-h dark photoperiod at 20 000 lux and 24°C served as the control. The tea plants were cut six times with scissors on the third leaf. Wounded leaves and the control were collected 48 h after wounding, immediately frozen in liquid nitrogen, and then stored at −80°C for further analysis.

For light treatment, pretreatment of tea seedlings was conducted by placing annual tea-cutting seedlings under fluorescent lamps for 2 days with a 14-h light/10-h dark photoperiod at 5000 lux and 24°C. Subsequently, the pretreated tea plants were placed in an environment with a light intensity of 60 000 lux as the experimental group, and the other conditions were unchanged. Tea seedlings in the pretreatment environment were used as controls. All samples were collected 48 h after treatment.

For MeJA and high-temperature treatments, annual tea cutting under fluorescent lamps with 14-h light/10-h dark photoperiod at 20 000 lux and 24°C served as pretreatment. Subsequently, the MeJA treatment group was sprayed with 100 μM MeJA and covered with a clear, closed mask. The high temperature treatment group was placed in a 40°C environment, and other conditions were unchanged. The MeJA control group was placed under pretreatment conditions and was sprayed with water. The high-temperature treatment control group was placed under pretreatment conditions. All samples were collected for gene expression determination 48 h after treatment.

*Arabidopsis* seedlings were used for various treatments 2 weeks after sowing. All *Arabidopsis* seedlings with the promoters of CsMYB5s were placed in an environment with a 14-h light/10-h dark photoperiod at 10 000 lux and 22°C and served as control. The treatment was conducted under high-intensity light, high temperature (40°C), mechanical wounding, MeJA, fungal infection, low temperature (4°C), 150 mM NaCl, 200 mM mannitol, 5 μM ABA, or 100 μM MeSA for 48 h and then collected for GUS staining.

### Transient overexpression and antisense interference of CsMYB5s

CsMYB5s were combined into the pCB2004 vector under the control of CaMV35S and then introduced into the *A. tumefaciens* GV3101 strain. The GV3101 strain containing pCB2004 empty vector and CsMYB5s were cultured to OD600 = 0.8 and collected by centrifugation at 5000 rpm for 7 min at room temperature. The supernatant was then removed and the precipitate was resuspended and the suspension centrifuged for collection. After repeating twice, the GV3101 strain containing CsMYB5s or pCB2004 was injected into the third leaf of tea plants. The injected tea leaves were collected for gene expression analysis and metabolic analysis after 72 h.

Candidate AsODNs of *CsMYB5b* and *CsMYB5f* were generated by Soligo software (https://pubmed.ncbi.nlm.nih.gov/31828806/) and the sODNs were used as control (Supplementary Data Table S4). The third leaves of tea plants were used for the AsODNexperiment and were injected with 100 μM AsODN or sODN. All samples were collected 48 h after treatment.

## Acknowledgements

This work was financially supported by the joint funds of National Natural Science Foundation of China (U21A20232), the Natural Science Foundation of China (32002088, 31870676, 32072621), and the National Key Research and Development Program of China (2018YFD1000601).

## Author contributions

Tianming Jiao, Liping Gao, and Tao Xia conceived and designed the experiments. Yipeng Huang, Yvchen Liu, Yajun Liu, and Xiaolan Jiang performed metabolic testing and analysis. Ting Jiang, Tongtong Li, Yanzhuo Liu, and Yunyun Han performed all experiments and analyzed the data. Tianming Jiao, Yingling Wu, Liping Gao, and Tao Xia wrote the manuscript. All authors approved the manuscript.

## Data availability

All data generated from the study appear in the submitted article.

## Conflicts of interest

The authors declare no competing interests

## Supplementary data

Supplementary data is available at *Horticulture Research* online.

## Supplementary Material

Web_Material_uhad135

## References

[1] Xie DY , Dixon RA. Proanthocyanidin biosynthesis – still more questions than answers? *Phytochemistry*. 2005;66:2127–44 16153412 10.1016/j.phytochem.2005.01.008

[2] Wang P , Liu Y, Zhang L et al. Functional demonstration of plant flavonoid carbocations proposed to be involved in the biosynthesis of proanthocyanidins. *Plant J*. 2020;101:18–36 31454118 10.1111/tpj.14515

[3] Zhuang J , Dai X, Zhu M et al. Evaluation of astringent taste of green tea through mass spectrometry-based targeted metabolic profiling of polyphenols. *Food Chem*. 2020;305:125507 31622805 10.1016/j.foodchem.2019.125507

[4] Zhao Z , Feng M, Wan J et al. Research progress of epigallocatechin-3-gallate (EGCG) on anti-pathogenic microbes and immune regulation activities. *Food Funct*. 2021;12:9607–19 34549212 10.1039/d1fo01352a

[5] Nichols JA , Katiyar SK. Skin photoprotection by natural polyphenols: anti-inflammatory, antioxidant and DNA repair mechanisms. *Arch Dermatol Res*. 2010;302:71–83 19898857 10.1007/s00403-009-1001-3 PMC2813915

[6] Fu Z , Jiang X, Li WW et al. Proanthocyanidin-aluminum complexes improve aluminum resistance and detoxification of *Camellia sinensis*. *J Agric Food Chem*. 2020;68:7861–9 32680420 10.1021/acs.jafc.0c01689

[7] Pfeiffer J , Kühnel C, Brandt J et al. Biosynthesis of catechins by leucoanthocyanidin 4-reductases (LAR) and anthocyanidin reductases (ANR) in leaves of grape (*Vitis vinifera*), apple (*Malus* x *domestica*) and other crops. *Plant Physiol Biochem*. 2006;44:323–34 16806954 10.1016/j.plaphy.2006.06.001

[8] Jiang X , Huang K, Zheng G et al. CsMYB5a and CsMYB5e from *Camellia sinensis* differentially regulate anthocyanin and proanthocyanidin biosynthesis. *Plant Sci*. 2018;270:209–20 29576074 10.1016/j.plantsci.2018.02.009

[9] Akagi T , Ikegami A, Tsujimoto T et al. DkMyb4 is a Myb transcription factor involved in proanthocyanidin biosynthesis in persimmon fruit. *Plant Physiol*. 2009;151:2028–45 19783643 10.1104/pp.109.146985 PMC2785967

[10] Bogs J , Jaffé FW, Takos AM et al. The grapevine transcription factor VvMYBPA1 regulates proanthocyanidin synthesis during fruit development. *Plant Physiol*. 2007;143:1347–61 17208963 10.1104/pp.106.093203 PMC1820911

[11] Mellway RD , Constabel CP. The wound-, pathogen-, and ultraviolet B-responsive *MYB134* gene encodes an R2R3 MYB transcription factor that regulates proanthocyanidin synthesis in poplar. *Plant Physiol*. 2009;150:924–41 19395405 10.1104/pp.109.139071 PMC2689947

[12] Liu C , Jun JH, Dixon RA. MYB5 and MYB14 play pivotal roles in seed coat polymer biosynthesis in *Medicago truncatula*. *Plant Physiol*. 2014;165:1424–39 24948832 10.1104/pp.114.241877 PMC4119029

[13] Hancock KR , Collette V, Fraser K et al. Expression of the R2R3-MYB transcription factor TaMYB14 from *Trifolium arvense* activates proanthocyanidin biosynthesis in the legumes *Trifolium repens* and *Medicago sativa*. *Plant Physiol*. 2012;159:1204–20 22566493 10.1104/pp.112.195420 PMC3387705

[14] Shen Y , Sun T, Pan Q et al. RrMYB5- and RrMYB10-regulated flavonoid biosynthesis plays a pivotal role in feedback loop responding to wounding and oxidation in *Rosa rugosa*. *Plant Biotechnol J*. 2019;17:2078–95 30951245 10.1111/pbi.13123 PMC6790370

[15] Nesi N , Jond C, Debeaujon I et al. The *Arabidopsis* TT2 gene encodes an R2R3 MYB domain protein that acts as a key determinant for proanthocyanidin accumulation in developing seed. *Plant Cell*. 2001;13:2099–114 11549766 10.1105/TPC.010098 PMC139454

[16] Deluc L , Bogs J, Walker AR et al. The transcription factor VvMYB5b contributes to the regulation of anthocyanin and proanthocyanidin biosynthesis in developing grape berries. *Plant Physiol*. 2008;147:2041–53 18539781 10.1104/pp.108.118919 PMC2492604

[17] Li SF , Milliken ON, Pham H et al. The *Arabidopsis* MYB5 transcription factor regulates mucilage synthesis, seed coat development, and trichome morphogenesis. *Plant Cell*. 2009;21:72–89 19136646 10.1105/tpc.108.063503 PMC2648076

[18] Quattrocchio F , Verweij W, Kroon A et al. PH4 of petunia is an R2R3 MYB protein that activates vacuolar acidification through interactions with basic-helix-loop-helix transcription factors of the anthocyanin pathway. *Plant Cell*. 2006;18:1274–91 16603655 10.1105/tpc.105.034041 PMC1456866

[19] James AM , Ma D, Mellway R et al. Poplar MYB115 and MYB134 transcription factors regulate proanthocyanidin synthesis and structure. *Plant Physiol*. 2017;174:154–71 28348066 10.1104/pp.16.01962 PMC5411147

[20] Karppinen K , Lafferty DJ, Albert NW et al. MYBA and MYBPA transcription factors co-regulate anthocyanin biosynthesis in blue-coloured berries. *New Phytol*. 2021;232:1350–67 34351627 10.1111/nph.17669

[21] Ullah C , Tsai CJ, Unsicker SB et al. Salicylic acid activates poplar defense against the biotrophic rust fungus *Melampsora larici-populina* via increased biosynthesis of catechin and proanthocyanidins. *New Phytol*. 2019;221:960–75 30168132 10.1111/nph.15396 PMC6585937

[22] Akagi T , Tsujimoto T, Ikegami A et al. Effects of seasonal temperature changes on DkMyb4 expression involved in proanthocyanidin regulation in two genotypes of persimmon (*Diospyros kaki* Thunb.) fruit. *Planta*. 2011;233:883–94 21225280 10.1007/s00425-010-1346-z

[23] Millard PS , Kragelund BB, Burow M. R2R3 MYB transcription factors - functions outside the DNA-binding domain. *Trends Plant Sci*. 2019;24:934–46 31358471 10.1016/j.tplants.2019.07.003

[24] Wang P , Ma G, Zhang L et al. A sucrose-induced MYB (SIMYB) transcription factor promoting proanthocyanidin accumulation in the tea plant (*Camellia sinensis*). *J Agric Food Chem*. 2019;67:1418–28 30688075 10.1021/acs.jafc.8b06207

[25] Zhao M , Zhang N, Gao T et al. Sesquiterpene glucosylation mediated by glucosyltransferase UGT91Q2 is involved in the modulation of cold stress tolerance in tea plants. *New Phytol*. 2020;226:362–72 31828806 10.1111/nph.16364

[26] Gonzalez A , Mendenhall J, Huo Y et al. TTG1 complex MYBs, MYB5 and TT2, control outer seed coat differentiation. *Dev Biol*. 2009;325:412–21 18992236 10.1016/j.ydbio.2008.10.005

[27] Liu Y , Hou H, Jiang X et al. A WD40 repeat protein from *Camellia sinensis* regulates anthocyanin and proanthocyanidin accumulation through the formation of MYB–bHLH–WD40 ternary complexes. *Int J Mol Sci*. 2018;19:1686 29882778 10.3390/ijms19061686 PMC6032167

[28] Solano R , Fuertes A, Sanchez-Pulido L et al. A single residue substitution causes a switch from the dual DNA binding specificity of plant transcription factor MYB.Ph3 to the animal c-MYB specificity. *J Biol Chem*. 1997;272:2889–95 9006933 10.1074/jbc.272.5.2889

[29] Stracke R , Turgut-Kara N, Weisshaar B. The AtMYB12 activation domain maps to a short C-terminal region of the transcription factor. *Z Naturforsch C J Biosci*. 2017;72:251–7 28284041 10.1515/znc-2016-0221

[30] Gibbs DJ , Voß U, Harding SA et al. AtMYB93 is a novel negative regulator of lateral root development in *Arabidopsis*. *New Phytol*. 2014;203:1194–207 24902892 10.1111/nph.12879 PMC4286813

[31] Dai X , Liu Y, Zhuang J et al. Discovery and characterization of tannase genes in plants: roles in hydrolysis of tannins. *New Phytol*. 2020;226:1104–16 32061142 10.1111/nph.16425

[32] An XH , Tian Y, Chen KQ et al. MdMYB9 and MdMYB11 are involved in the regulation of the JA-induced biosynthesis of anthocyanin and proanthocyanidin in apples. *Plant Cell Physiol*. 2015;56:650–62 25527830 10.1093/pcp/pcu205

[33] Wang N , Qu C, Jiang S et al. The proanthocyanidin-specific transcription factor MdMYBPA1 initiates anthocyanin synthesis under low-temperature conditions in red-fleshed apples. *Plant J*. 2018;96:39–55 29978604 10.1111/tpj.14013

[34] Li Y , Shan X, Zhou L et al. The R2R3-MYB factor FhMYB5 from *Freesia hybrida* contributes to the regulation of anthocyanin and proanthocyanidin biosynthesis. *Front Plant Sci*. 2019;9:1935 30666265 10.3389/fpls.2018.01935 PMC6330306

[35] Tanikawa J , Yasukawa T, Enari M et al. Recognition of specific DNA sequences by the *c-myb* protooncogene product: role of three repeat units in the DNA-binding domain. *Proc Natl Acad Sci USA*. 1993;90:9320–4 8415700 10.1073/pnas.90.20.9320 PMC47559

[36] Koyama K , Ikeda H, Poudel PR et al. Light quality affects flavonoid biosynthesis in young berries of Cabernet Sauvignon grape. *Phytochemistry*. 2012;78:54–64 22455871 10.1016/j.phytochem.2012.02.026

[37] Li C , Pei J, Yan X et al. A poplar B-box protein PtrBBX23 modulates the accumulation of anthocyanins and proanthocyanidins in response to high light. *Plant Cell Environ*. 2021;44:3015–33 34114251 10.1111/pce.14127

[38] Mao Z , Jiang H, Wang S et al. The MdHY5-MdWRKY41-MdMYB transcription factor cascade regulates the anthocyanin and proanthocyanidin biosynthesis in red-fleshed apple. *Plant Sci*. 2021;306:110848 33775373 10.1016/j.plantsci.2021.110848

[39] An J-P , Xu RR, Liu X et al. Jasmonate induces anthocyanin and proanthocyanidin biosynthesis in apple by mediating the JAZ1-TRB1-MYB9 complex. *Plant J*. 2021;106:1414–30 33759251 10.1111/tpj.15245

[40] Wang S , Li LX, Fang Y et al. MdERF1B-MdMYC2 module integrates ethylene and jasmonic acid to regulate the biosynthesis of anthocyanin in apple. *Hortic Res*. 2022;9:142 10.1093/hr/uhac142 PMC9437725 36072842

[41] Cheong YH , Chang HS, Gupta R et al. Transcriptional profiling reveals novel interactions between wounding, pathogen, abiotic stress, and hormonal responses in *Arabidopsis*. *Plant Physiol*. 2002;129:661–77 12068110 10.1104/pp.002857 PMC161692

[42] Takashi A , Ayako IK, Yonemori K. DkMYB2 wound-induced transcription factor of persimmon (*Diospyros kaki* Thunb.), contributes to proanthocyanidin regulation. *Planta*. 2010;232:1045–59 20690029 10.1007/s00425-010-1241-7

[43] Zhu Q , Sui S, Lei X et al. Ectopic expression of the coleus R2R3 MYB-type proanthocyanidin regulator gene *SsMYB3* alters the flower color in transgenic tobacco. *PLoS One*. 2015;10:e0139392 26448466 10.1371/journal.pone.0139392 PMC4598174

