# Supplementary material for: Functional diversity of subgroup 5 R2R3-MYBs promoting proanthocyanidin biosynthesis and their key residues and motifs in tea plant
Source: Hortic Res. 2023 Jul 5;10(8):uhad135. doi: 10.1093/hr/uhad135 (PMC10484168; doi:10.1093/hr/uhad135)
Supplement: Web_Material_uhad135 [file web_material_uhad135.doc]

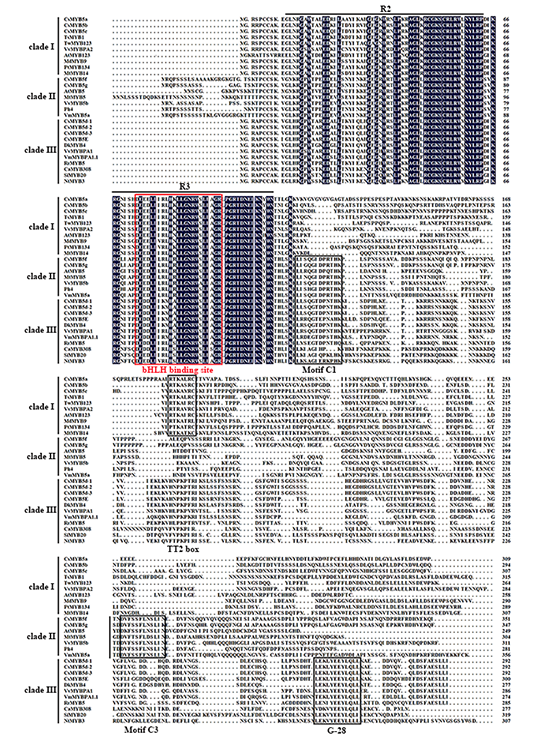


Figure S1. Protein alignment among three types of MYB5 in different species.

Full length amino acid sequences of CsMYB5s in *Camellia sinensis* and subgroup 5 R2R3-MYBs in other plants were used for amino acid sequence alignment using the DNAMAN program (Lynnon Corporation, San Ramon, CA, USA). R2 and R3 domains were marked with a black line. The bHLH motifs were marked by a red box. The special motifs of three clades of MYB5s were marked by black boxes.


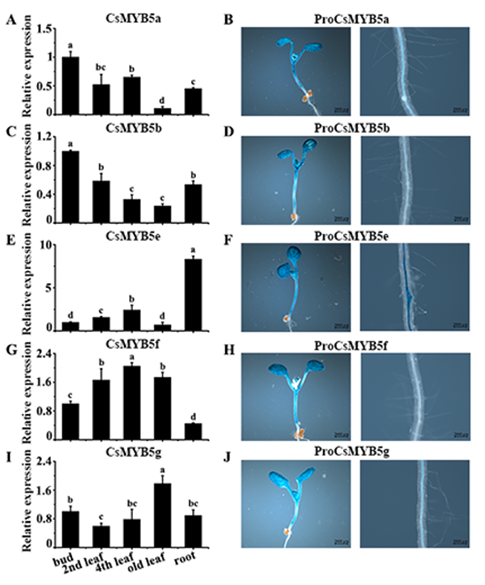


Figure S2. Tissue-specificity gene expression of CsMYB5s.

(A, C, E, G and I) qRT-PCR analysis of mRNA level of CsMYB5s in tissues of bud, 2nd leaf, 4th leaf, old leaf and root. Means were calculated from three repeats and error bars reflect ±SDs. The different letters mean the significance level at p < 0.05 based on a Tukey’s honestly significant difference test (n=3). (B, D, F, H and J) GUS expression pattern in the aerial parts and root of proMYB5s:GUS reporter lines. The scale bars indicate 2 mm


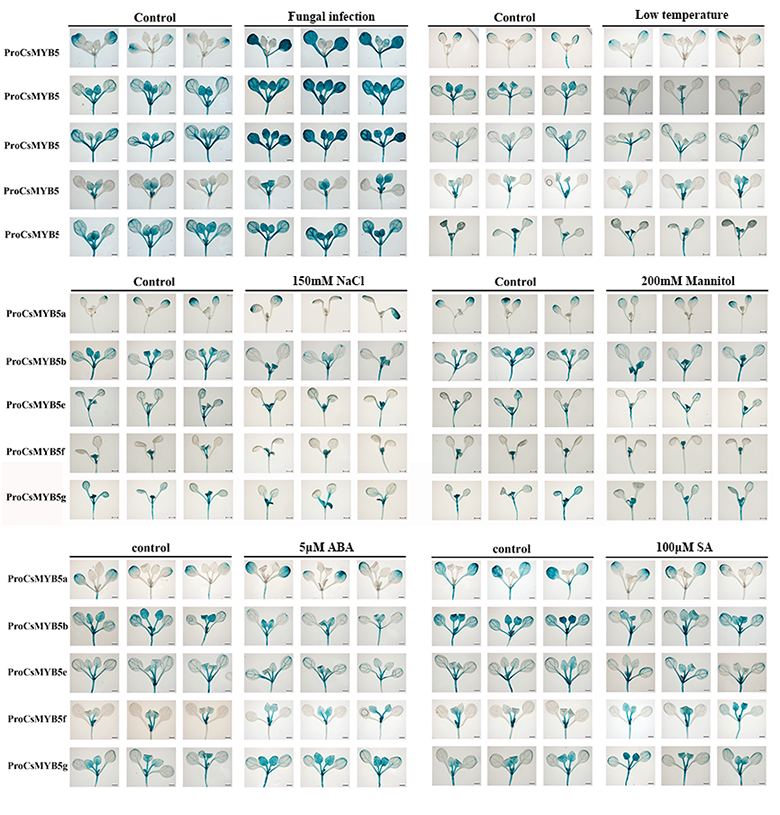


Figure S3. The promoter viability analysis of CsMYB5s under a series of treatments

The histochemical assay of the promoters of CsMYB5s under a series of treatments such as fungal infection, low temperature (4°C), salt (150 mM NaCl), 200 mM mannitol, 5 µM ABA, and 100 µM MeSA, respectively, for 48 h. All control arabidopsis seedlings were cultured in an environment with 14-h light/10-h dark photoperiod at 10000 Lux at 22 °C. The scale bars indicate 2 mm.


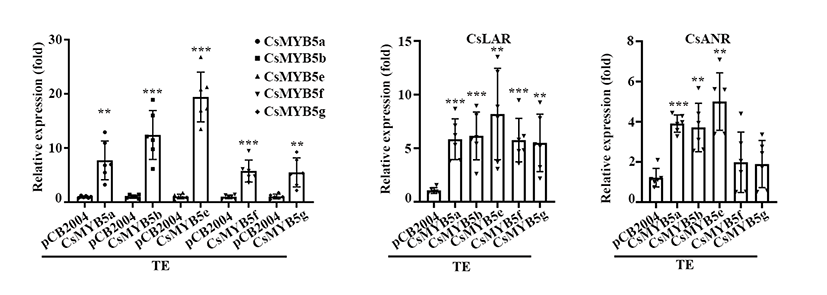


Figure S4. Transient overexpression of CsMYB5s in tea plant leaves

qRT-PCR analysis of mRNA level of *CsMYB5a/b/e/f/g*, *CsLAR*, and *CsANR* in the tea plant leaves that transiently overexpressed CsMYB5s. Means were calculated from six repeats and error bars reflect ±SDs. Asterisks indicate significant differences using Student’s *t*-test (n=6, **p* < 0.05, ***p* < 0.01, ****p* < 0.001).


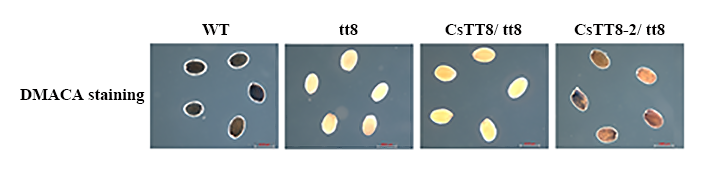


Figure S5. DMACA staining in mature arabidopsis seeds of WT, *tt8*, and *CsTT8* and *CsTT8-2* complementary *tt8*


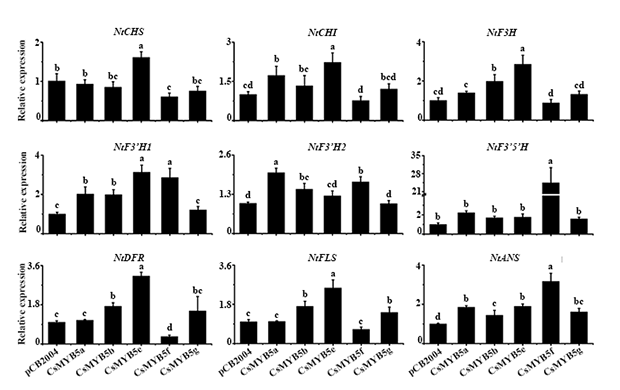


Figure S6. Gene expression of flavonoid pathway in CsMYB5s transgenic tobacco.

CHS, chalcone synthase; CHI, chalcone isomerase; F3H, flavonoid 3-hydroxylase; F3′H, flavonoid 3′-hydroxylase; F3’5’H, flavonoid 3’5’-hydroxylase; DFR, dihydroflavanol reductase; FLS, flavonol synthase; ANS, anthocyanidin synthase. All qRT-PCR analyses were performed on three independent biological replicates, giving technical triplicates. The different letters mean the significance level at *p* < 0.05 based on a Tukey’s honestly significant difference test (n=3).


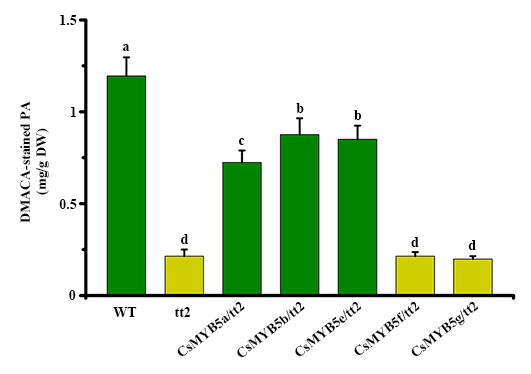


Figure S7 The content of DMACA-stained PA in CsMYB5s transgenic *tt2* mutant

All data are the means of three biological replicates. Different letters indicate significant differences among group at α = 0.05 as determined by Duncan's multiple range test (n=3). DW, dry weight.


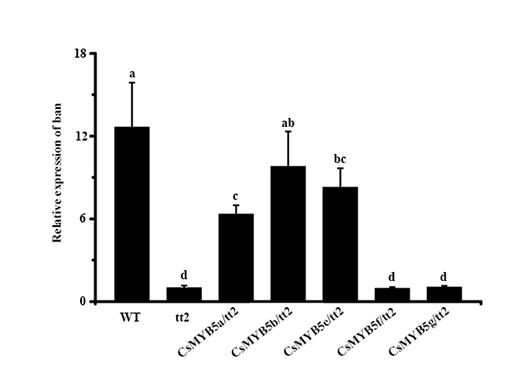


Figure S8 The gene expression of *AtBAN* in CsMYB5s transgenic *tt2* mutant

All the values are the means of three biological replicates, and the error bars represent the standard deviation of three replicates. Different letters indicate significant differences among group at α = 0.05 as determined by Duncan's multiple range test (n=3).


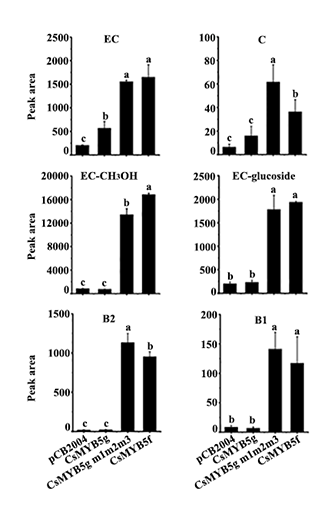


Fig S9 Accumulation of polyphenol metabolites in CsMYB5f/5g/5g m1m2m3 transgenic tobacco flowers

Related content of EC-CH_3_OH (m/z 319), C (m/z 289), PAs B1 (m/z 577), EC-Glu (m/z 451), EC (m/z 289) and PAs B2 (m/z 577), in control and CsMYB5s-overexpressing tobacco flowers analyzed through MS-based multiple reaction monitoring. All data are the means of three biological replicates. and the error bars denote standard deviation. The different letters mean the significance level at *p* < 0.05 based on a Tukey’s honestly significant difference test (n=3).


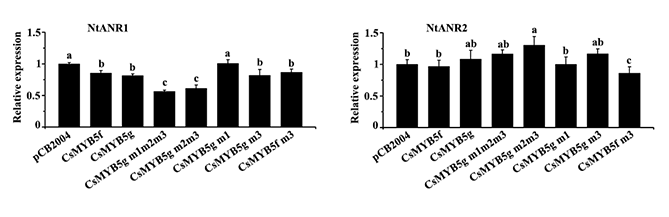


Fig S10 The gene expression of *NtANR1* and *NtANR2* in *CsMYB5f/g* and point mutated *CsMYB5f/g* transgenic tobacco flowers

Relative expression of *NtANR1* and *NtANR2* in transgenic tobacco flowers through qRT-PCR. All data are the means of three biological replicates. and the error bars denote standard deviation. The different letters mean the significance level at *p* < 0.05 based on a Tukey’s honestly significant difference test (n=3).


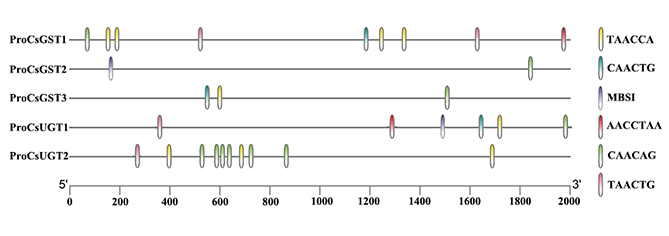


Fig S11 Schematic representation of the promoter cis-acting elements of *CsGSTs* and *CsUGTs*

The promoter information of *CsGSTs* and *CsUGTs* were obtained from the genome of *Tieguanyin* and the promoter cis-acting elements were predicted with the online software (PlantCARE). Schematic representation of the promoters were performed by TBtools.


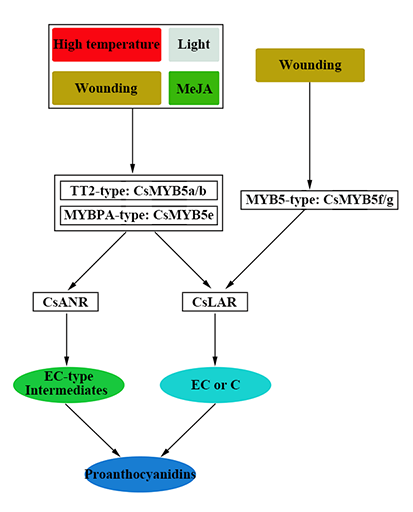


Figure S12. Schematic model of CsMYB5s involved in PA biosynthesis.

TT2-type and MYBPA-type were induced by high-intensity light, high temperature, MeJA, and mechanical wounding, and promoted the PA biosynthesis by regulating *CsLAR* and *CsANR* in tea plant. However, MYB5-type was only induced by mechanical wounding and promoted PA biosynthesis by regulating *CsLAR*, while MYB5-type could not activate the expression of *CsANR.* Previous studies have shown that *CsANR* is responsible for the formation of epicatechin intermediates, and *CsLAR* is responsible for the formation of catechin EC and C. Catechins (EC and C) and the intermediates undergo polymerization reaction to produce proanthocyanidins. The solid arrows indicate findings supported by experimental evidence obtained in this study.

Table S1

| (MYB)gene ID | (MYB)gene ID | (MYB)gene ID | (MYB)gene ID | (MYB)gene ID |
| --- | --- | --- | --- | --- |
| TGY000597.t1 | TGY008059.t1 | TGY010454.t1 | TGY040181.t1 | TGY067551.t1 |
| TGY001891.t1 | TGY008588.t1 | TGY010470.t1 | TGY040359.t1 | TGY070068.t1 |
| TGY002304.t1 | TGY008649.t1 | TGY010480.t1 | TGY041582.t1 | TGY070180.t1 |
| TGY003575.t1 | TGY008659.t1 | TGY010569.t1 | TGY041768.t1 | TGY070260.t1 |
| TGY003634.t1 | TGY008811.t1 | TGY010580.t1 | TGY044528.t1 | TGY072552.t1 |
| TGY004382.t1 | TGY009071.t1 | TGY010783.t1 | TGY045089.t1 | TGY074519.t1 |
| TGY004410.t1 | TGY010027.t1 | TGY011773.t1 | TGY045091.t1 | TGY074569.t1 |
| TGY005981.t1 | TGY010089.t1 | TGY012052.t1 | TGY045119.t1 | TGY075924.t1 |
| TGY006538.t1 | TGY010092.t1 | TGY012231.t1 | TGY048165.t1 | TGY076520.t1 |
| TGY007121.t1 | TGY010305.t1 | TGY012479.t1 | TGY048360.t1 | TGY109715.t1 |
| TGY012624.t1 | TGY021855.t1 | TGY012519.t1 | TGY050097.t1 | TGY110225.t1 |
| TGY012802.t1 | TGY022161.t1 | TGY012529.t1 | TGY051273.t1 | TGY111163.t1 |
| TGY013828.t1 | TGY027188.t1 | TGY078694.t1 | TGY051921.t1 | TGY102924.t1 |
| TGY014154.t1 | TGY030717.t1 | TGY078696.t1 | TGY052718.t1 | TGY103859.t1 |
| TGY014213.t1 | TGY030718.t1 | TGY078897.t1 | TGY053365.t1 | TGY104129.t1 |
| TGY014249.t1 | TGY030720.t1 | TGY081981.t1 | TGY053369.t1 | TGY105015.t1 |
| TGY014667.t1 | TGY030990.t1 | TGY082692.t1 | TGY054228.t1 | TGY105021.t1 |
| TGY014924.t1 | TGY031421.t1 | TGY087224.t1 | TGY054465.t1 | TGY105274.t1 |
| TGY014952.t1 | TGY031902.t1 | TGY087655.t1 | TGY055016.t1 | TGY105348.t1 |
| TGY015059.t1 | TGY032218.t1 | TGY089884.t1 | TGY055852.t1 | TGY105531.t1 |
| TGY019964.t1 | TGY032479.t1 | TGY090900.t1 | TGY058405.t1 | TGY105797.t1 |
| TGY020064.t1 | TGY033028.t1 | TGY091189.t1 | TGY059315.t1 | TGY105932.t1 |
| TGY111227.t1 | TGY033869.t1 | TGY091649.t1 | TGY062271.t1 | TGY077108.t1 |
| TGY111248.t1 | TGY036243.t1 | TGY093965.t1 | TGY062275.t1 | TGY077260.t1 |
| TGY111267.t1 | TGY036382.t1 | TGY094582.t1 | TGY062281.t1 | TGY077414.t1 |
| TGY111379.t1 | TGY038644.t1 | TGY095416.t1 | TGY062300.t1 | TGY078684.t1 |
| TGY111380.t1 | TGY039742.t1 | TGY097786.t1 | TGY062315.t1 | TGY078689.t1 |
| TGY114015.t1 | TGY076554.t1 | TGY098839.t1 | TGY063605.t1 | TGY124062.t1 |
| TGY114022.t1 | TGY076645.t1 | TGY098872.t1 | TGY063628.t1 | TGY124380.t1 |
| TGY114709.t1 | TGY076670.t1 | TGY102791.t1 | TGY067542.t1 | TGY124556.t1 |
| TGY114973.t1 | TGY076914.t1 | TGY102821.t1 | TGY067543.t1 | TGY126086.t1 |
| TGY115853.t1 | TGY117284.t1 | TGY120119.t1 | TGY121353.t1 | TGY126479.t1 |
| TGY116729.t1 | TGY119466.t1 | TGY121332.t1 | TGY121471.t1 | TGY108773.t1 |
| TGY108814.t1 |  |  |  |  |

Table S2

| *Vitis vinifera* | | | | | | | | |
| --- | --- | --- | --- | --- | --- | --- | --- | --- |
| VvMYBPA1-CAJ90831 | | | VvMYB5b-AAX51291.1 | | | VvMYBPA2-ACK56131 | | |
| Alignments | Score | E-Value | Alignments | Score | E-Value | Alignments | Score | E-Value |
| TGY030718.t1 | 365 | 1E-104 | TGY111380.t1 | 360 | 1E-102 | TGY121353.t1 | 251 | 2E-69 |
| TGY030720.t1 | 363 | 1E-103 | TGY111379.t1 | 358 | 1E-102 | TGY120119.t1 | 241 | 1E-66 |
| TGY030717.t1 | 361 | 1E-102 | TGY030718.t1 | 225 | 1E-61 | TGY114022.t1 | 236 | 6E-65 |
| TGY054465.t1 | 337 | 3E-95 | TGY030720.t1 | 224 | 2E-61 | TGY030718.t1 | 209 | 1E-56 |
| TGY111379.t1 | 225 | 1E-61 | TGY030717.t1 | 223 | 5E-61 | TGY030720.t1 | 208 | 2E-56 |
| TGY059315.t1 | 221 | 3E-60 | TGY054465.t1 | 220 | 5E-60 | TGY030717.t1 | 208 | 2E-56 |
| TGY111380.t1 | 220 | 5E-60 | TGY055016.t1 | 214 | 4E-58 | TGY054465.t1 | 204 | 3E-55 |
| TGY077108.t1 | 218 | 2E-59 | TGY121353.t1 | 204 | 2E-55 | TGY093965.t1 | 192 | 1E-51 |
| TGY093965.t1 | 217 | 3E-59 | TGY036382.t1 | 202 | 9E-55 | TGY111380.t1 | 191 | 2E-51 |
| TGY031421.t1 | 216 | 5E-59 | TGY031421.t1 | 201 | 2E-54 | TGY111379.t1 | 191 | 3E-51 |
| TGY120119.t1 | 216 | 9E-59 | TGY074519.t1 | 200 | 6E-54 | TGY087655.t1 | 189 | 1E-50 |
| TGY121353.t1 | 214 | 3.00E-58 | TGY014154.t1 | 199 | 7E-54 | TGY014154.t1 | 189 | 1E-50 |
| TGY036382.t1 | 213 | 4.00E-58 | TGY070260.t1 | 199 | 9E-54 | TGY036382.t1 | 187 | 4E-50 |
| TGY114022.t1 | 212 | 1.00E-57 | TGY116729.t1 | 198 | 2E-53 | TGY031421.t1 | 186 | 1E-49 |
| TGY012479.t1 | 206 | 9.00E-56 | TGY114022.t1 | 197 | 3E-53 | TGY077108.t1 | 185 | 2E-49 |
| *Vaccinium myrtillus* | | | | | | | | |
| VmMYBPA1.1-QWW89542 | | | VmMYB5a-MT316029 | | | VmMYBPA2.1-QWW89544 | | |
| Alignments | Score | E-Value | Alignments | Score | E-Value | Alignments | Score | E-Value |
| TGY030718.t1 | 370 | 1E-105 | TGY111379.t1 | 394 | 1E-112 | TGY121353.t1 | 358 | 1E-102 |
| TGY030720.t1 | 370 | 1E-105 | TGY111380.t1 | 379 | 1E-108 | TGY120119.t1 | 266 | 5E-74 |
| TGY030717.t1 | 367 | 1E-104 | TGY030720.t1 | 232 | 2E-63 | TGY114022.t1 | 241 | 3E-66 |
| TGY054465.t1 | 358 | 1E-101 | TGY030718.t1 | 230 | 4E-63 | TGY030720.t1 | 209 | 7E-57 |
| TGY111379.t1 | 229 | 1E-62 | TGY030717.t1 | 230 | 4E-63 | TGY030718.t1 | 209 | 7E-57 |
| TGY111380.t1 | 225 | 1E-61 | TGY054465.t1 | 224 | 4E-61 | TGY030717.t1 | 209 | 1E-56 |
| TGY121353.t1 | 218 | 1E-59 | TGY055016.t1 | 209 | 1E-56 | TGY054465.t1 | 207 | 3E-56 |
| TGY120119.t1 | 214 | 2E-58 | TGY036382.t1 | 204 | 3E-55 | TGY111379.t1 | 197 | 3E-53 |
| TGY114022.t1 | 210 | 4E-57 | TGY074519.t1 | 203 | 6E-55 | TGY111380.t1 | 196 | 8E-53 |
| TGY077108.t1 | 209 | 6E-57 | TGY031421.t1 | 203 | 6E-55 | TGY014154.t1 | 192 | 9E-52 |
| TGY059315.t1 | 209 | 8E-57 | TGY087655.t1 | 202 | 1E-54 | TGY036382.t1 | 192 | 1E-51 |
| TGY031421.t1 | 209 | 1E-56 | TGY070260.t1 | 200 | 5E-54 | TGY093965.t1 | 191 | 2E-51 |
| TGY036382.t1 | 205 | 1E-55 | TGY077108.t1 | 198 | 2E-53 | TGY077108.t1 | 191 | 2E-51 |
| TGY014154.t1 | 202 | 7E-55 | TGY014154.t1 | 198 | 2E-53 | TGY059315.t1 | 191 | 2E-51 |
| TGY093965.t1 | 202 | 1E-54 | TGY010027.t1 | 198 | 2E-53 | TGY012479.t1 | 191 | 2E-51 |
| *Populus trichocarpa* | | | | | | | | |
| PtMYB115-XP_002302644 | | | PtMYB5-XP_002297634 | | | PtMYB134-ACR83705 | | |
| Alignments | Score | E-Value | Alignments | Score | E-Value | Alignments | Score | E-Value |
| TGY030718.t1 | 364 | 1E-103 | TGY111379.t1 | 332 | 1E-93 | TGY114022.t1 | 276 | 6E-77 |
| TGY030717.t1 | 362 | 1E-103 | TGY111380.t1 | 330 | 3E-93 | TGY120119.t1 | 266 | 6E-74 |
| TGY030720.t1 | 359 | 1E-102 | TGY030718.t1 | 223 | 6E-61 | TGY121353.t1 | 256 | 5E-71 |
| TGY054465.t1 | 337 | 3E-95 | TGY030720.t1 | 223 | 8E-61 | TGY030717.t1 | 210 | 4E-57 |
| TGY111380.t1 | 229 | 6E-63 | TGY030717.t1 | 223 | 8E-61 | TGY030720.t1 | 209 | 8E-57 |
| TGY111379.t1 | 228 | 2E-62 | TGY054465.t1 | 215 | 2E-58 | TGY030718.t1 | 209 | 8E-57 |
| TGY077108.t1 | 213 | 4E-58 | TGY055016.t1 | 214 | 4E-58 | TGY054465.t1 | 207 | 3E-56 |
| TGY031421.t1 | 210 | 5E-57 | TGY036382.t1 | 204 | 2E-55 | TGY111380.t1 | 204 | 4E-55 |
| TGY114022.t1 | 209 | 6E-57 | TGY012052.t1 | 204 | 4E-55 | TGY093965.t1 | 202 | 1E-54 |
| TGY059315.t1 | 209 | 6E-57 | TGY010027.t1 | 202 | 9E-55 | TGY111379.t1 | 197 | 3E-53 |
| TGY120119.t1 | 208 | 1E-56 | TGY074519.t1 | 202 | 1E-54 | TGY077108.t1 | 195 | 2E-52 |
| TGY036382.t1 | 206 | 5E-56 | TGY077108.t1 | 202 | 1E-54 | TGY059315.t1 | 192 | 1E-51 |
| TGY121353.t1 | 206 | 7E-56 | TGY070260.t1 | 202 | 1E-54 | TGY036382.t1 | 192 | 1E-51 |
| TGY093965.t1 | 205 | 2E-55 | TGY059315.t1 | 201 | 3E-54 | TGY008588.t1 | 188 | 2E-50 |
| TGY055016.t1 | 202 | 8E-55 | TGY014154.t1 | 201 | 3E-54 | TGY031421.t1 | 187 | 4E-50 |
| *Diospyros kaki* | | | *Arabidopsis thaliana* | | | | | |
| DkMYB4-BAI49721 | | | AtMYB5-NP_187963 | | | AtMYB123-CAC40021 | | |
| Alignments | Score (bits) | E-Value | Alignments | Score (bits) | E-Value | Alignments | Score (bits) | E-Value |
| TGY030718.t1 | 404 | 1E-115 | TGY111380.t1 | 255 | 9E-71 | TGY120119.t1 | 199 | 6E-54 |
| TGY030720.t1 | 400 | 1E-114 | TGY111379.t1 | 249 | 8E-69 | TGY121353.t1 | 195 | 1E-52 |
| TGY030717.t1 | 399 | 1E-114 | TGY030720.t1 | 219 | 5E-60 | TGY114022.t1 | 183 | 6E-49 |
| TGY054465.t1 | 362 | 1E-103 | TGY030718.t1 | 219 | 5E-60 | TGY030718.t1 | 175 | 2E-46 |
| TGY111379.t1 | 224 | 3E-61 | TGY030717.t1 | 216 | 4E-59 | TGY105348.t1 | 174 | 3E-46 |
| TGY111380.t1 | 223 | 5E-61 | TGY054465.t1 | 216 | 6E-59 | TGY030720.t1 | 172 | 8E-46 |
| TGY036382.t1 | 212 | 1E-57 | TGY055016.t1 | 204 | 2E-55 | TGY008588.t1 | 172 | 8E-46 |
| TGY121353.t1 | 211 | 2E-57 | TGY036382.t1 | 203 | 4E-55 | TGY030717.t1 | 172 | 1E-45 |
| TGY120119.t1 | 211 | 2E-57 | TGY077108.t1 | 203 | 5E-55 | TGY087655.t1 | 171 | 2E-45 |
| TGY077108.t1 | 211 | 2E-57 | TGY031421.t1 | 201 | 2E-54 | TGY124556.t1 | 171 | 2E-45 |
| TGY031421.t1 | 211 | 3E-57 | TGY059315.t1 | 201 | 2E-54 | TGY012479.t1 | 171 | 2E-45 |
| TGY059315.t1 | 210 | 4E-57 | TGY074519.t1 | 200 | 3E-54 | TGY054465.t1 | 171 | 3E-45 |
| TGY093965.t1 | 209 | 1E-56 | TGY014154.t1 | 200 | 4E-54 | TGY014154.t1 | 171 | 3E-45 |
| TGY114022.t1 | 208 | 1E-56 | TGY070260.t1 | 199 | 6E-54 | TGY014924.t1 | 170 | 5E-45 |
| TGY014154.t1 | 205 | 2E-55 | TGY062315.t1 | 196 | 8E-53 | TGY111380.t1 | 169 | 1E-44 |
| *Rosa rugosa* | | | *Medicago truncatula* | | | | | |
| RrMYB5-AYP10274 | | | MtMYB5-XP_003601609.2 | | | MtMYB14-XP_013458423 | | |
| Alignments | Score (bits) | E-Value | Alignments | Score (bits) | E-Value | Alignments | Score (bits) | E-Value |
| TGY030717.t1 | 348 | 1E-98 | TGY111379.t1 | 287 | 3E-80 | TGY120119.t1 | 273 | 4E-76 |
| TGY030718.t1 | 346 | 6E-98 | TGY111380.t1 | 281 | 2E-78 | TGY121353.t1 | 264 | 3E-73 |
| TGY030720.t1 | 342 | 1E-96 | TGY030720.t1 | 226 | 7E-62 | TGY114022.t1 | 254 | 3E-70 |
| TGY054465.t1 | 323 | 5E-91 | TGY030718.t1 | 226 | 7E-62 | TGY030718.t1 | 216 | 7E-59 |
| TGY111379.t1 | 231 | 3E-63 | TGY030717.t1 | 226 | 7E-62 | TGY030720.t1 | 214 | 2E-58 |
| TGY111380.t1 | 229 | 6E-63 | TGY054465.t1 | 218 | 2E-59 | TGY030717.t1 | 214 | 4E-58 |
| TGY077108.t1 | 218 | 2E-59 | TGY055016.t1 | 209 | 1E-56 | TGY054465.t1 | 207 | 4E-56 |
| TGY031421.t1 | 216 | 7E-59 | TGY031421.t1 | 207 | 4E-56 | TGY111380.t1 | 199 | 7E-54 |
| TGY120119.t1 | 215 | 1E-58 | TGY120119.t1 | 205 | 2E-55 | TGY014154.t1 | 199 | 7E-54 |
| TGY059315.t1 | 215 | 1E-58 | TGY077108.t1 | 204 | 2E-55 | TGY093965.t1 | 197 | 4E-53 |
| TGY114022.t1 | 214 | 3E-58 | TGY114022.t1 | 204 | 4E-55 | TGY111379.t1 | 195 | 2E-52 |
| TGY121353.t1 | 214 | 3E-58 | TGY121353.t1 | 203 | 5E-55 | TGY008588.t1 | 192 | 1E-51 |
| TGY036382.t1 | 210 | 4E-57 | TGY059315.t1 | 202 | 1E-54 | TGY036382.t1 | 191 | 3E-51 |
| TGY093965.t1 | 210 | 5E-57 | TGY036382.t1 | 202 | 1E-54 | TGY062315.t1 | 190 | 4E-51 |
| TGY051273.t1 | 207 | 2E-56 | TGY074519.t1 | 201 | 2E-54 | TGY059315.t1 | 190 | 4E-51 |
| gene ID | name | | |  |  |  |  |  |
| TGY121353.t1 | CsMYB5a | | |  |  |  |  |  |
| TGY120119.t1 | CsMYB5b | | |  |  |  |  |  |
| TGY114022.t1 | CsMYB5c | | |  |  |  |  |  |
| TGY030718.t1 | CsMYB5d-1 | | |  |  |  |  |  |
| TGY030720.t1 | CsMYB5d-2 | | |  |  |  |  |  |
| TGY030717.t1 | CsMYB5d-3 | | |  |  |  |  |  |
| TGY054465.t1 | CsMYB5e | | |  |  |  |  |  |
| TGY111380.t1 | CsMYB5f | | |  |  |  |  |  |
| TGY111379.t1 | CsMYB5g | | |  |  |  |  |  |

Table S3

| Angiosperms classification | Species | MYB5 classification | Gene name | Gene ID | Chr | Start | End |
| --- | --- | --- | --- | --- | --- | --- | --- |
| Dicoty  ledons | *Camellia*  *sinensis* | TT2 type | CsMYB5a | GWHPAZTZ039135 | Chromosome 14 | 5381295 | 5382482 |
|  |  |  | CsMYB5b | GWHPAZTZ041781 | Chromosome 15 | 18263647 | 18266842 |
|  |  |  | CsMYB5c | GWHPAZTZ019010 | Chromosome 6 | 89844738 | 89846647 |
|  |  | MYBPA type | CsMYB5d | GWHPAZTZ010324 | Chromosome 3 | 207486404 | 207488582 |
|  |  |  | CsMYB5d | GWHPAZTZ010326 | Chromosome 3 | 207496213 | 207515637 |
|  |  |  | CsMYB5d | GWHPAZTZ010328 | Chromosome 3 | 207569340 | 207580262 |
|  |  |  | CsMYB5e | GWHPAZTZ041490 | Chromosome 14 | 147329781 | 147332570 |
|  |  | MYB5 type | CsMYB5f | GWHPAZTZ038246 | Chromosome 13 | 115970557 | 115971867 |
|  |  |  | CsMYB5g | GWHPAZTZ038247 | Chromosome 13 | 115989899 | 115991387 |
|  | Vaccinium  darrowii | TT2 type | TT2-1 | KAH7858361 | chromosome 3 | 46154619 | 46156499 |
|  |  |  | TT2-2 | KAH7856222 | chromosome 11 | 5556519 | 5558316 |
|  |  |  | TT2-3 | KAH7865697 | chromosome 9 | 42768063 | 42769262 |
|  |  |  | TT2-4 | KAH7853118 | chromosome 8 | 42633963 | 42636323 |
|  |  |  | TT2-5 | KAH7851587 | chromosome 8 | 42600154 | 42602729 |
|  |  | MYBPA type | MYBPA-1 | KAH7864802 | chromosome 12 | 585708 | 586936 |
|  |  |  | MYBPA-2 | KAH7861755 | chromosome 4 | 27721642 | 27723451 |
|  |  | MYB5 type | MYB5-1 | KAH7837513 | chromosome 6 | 2220544 | 2221976 |
|  |  |  | MYB5-2 | KAH7859850 | chromosome 4 | 25571946 | 25573144 |
|  | *Actinidia chinensis*  *var. chinensis* | TT2 type | TT2-1 | PSS11750 | LG14 | 10860154 | 10861485 |
|  |  |  | TT2-2 | PSR96942 | LG23 | 27131897 | 27133675 |
|  |  |  | TT2-3 | PSS20780 | LG9 | 789519 | 791553 |
|  |  |  | TT2-4 | PSS36408 | LG1 | 14514361 | 14516347 |
|  |  |  | TT2-5 | PSR96197 | LG23 | 18928301 | 18930220 |
|  |  |  | TT2-6 | PSS15475 | LG12 | 735690 | 738123 |
|  |  |  | TT2-7 | PSS18100 | LG11 | 14839874 | 14846002 |
|  |  | MYBPA type | MYBPA-1 | PSS23525 | LG8 | 477539 | 478924 |
|  |  |  | MYBPA-2 | PSS03927 | LG18 | 376317 | 377706 |
|  |  | MYB5 type | MYB5-1 | PSS14497 | LG13 | 16084710 | 16086164 |
|  |  |  | MYB5-2 | PSR95360 | LG23 | 9371063 | 9372574 |
|  |  |  | MYB5-3 | PSR86903 | LG28 | 15415840 | 15417493 |
|  | *Vitis*  *vinifera* | TT2 type | TT2-1 | LOC100264614 | Chromosome 4 | 1418688 | 1420514 |
|  |  |  | TT2-2 | LOC100254224 | Chromosome 11 | 1044483 | 1045909 |
|  |  |  | TT2-3 | LOC100244051 | Chromosome 4 | 1456153 | 1457851 |
|  |  |  | TT2-4 | LOC100260951 | Chromosome 13 | 19507330 | 19508302 |
|  |  |  | TT2-5 | LOC100250940 | Chromosome 11 | 1055702 | 1057696 |
|  |  | MYBPA type | MYBPA1 | LOC100232899 | Chromosome 15 | 17211667 | 17213117 |
|  |  |  | MYBPA2 | LOC100261618 | Chromosome 2 | 1964746 | 1966004 |
|  |  | MYB5 type | MYB5-1 | LOC100232973 | Chromosome 6 | 744553 | 745906 |
|  |  |  | MYB5-2 | LOC100233122 | Chromosome 8 | 20865379 | 20866669 |
|  | *Theobroma*  *cacao* | TT2 type | TT2-1 | LOC18588141 | Chromosome 9 | 3096888 | 3098447 |
|  |  |  | TT2-2 | LOC18596211 | Chromosome 6 | 19896827 | 19898583 |
|  |  |  | TT2-3 | LOC18588308 | Chromosome 9 | 4143603 | 4145643 |
|  |  |  | TT2-4 | LOC18588305 | Chromosome 9 | 4111054 | 4112659 |
|  |  | MYBPA type | MYBPA | LOC18613948 | Chromosome 1 | 33467854 | 33469310 |
|  |  | MYB5 type | MYB5-1 | LOC18598703 | Chromosome 5 | 22204639 | 22206418 |
|  |  |  | MYB5-2 | LOC18599853 | Chromosome 5 | 34601360 | 34603264 |
|  | *Solanum*  *lycopersicum* | TT2 type | TT2-1 | LOC101264905 | Chromosome 12 | 61725446 | 61727515 |
|  |  | MYBPA type | MYBPA-1 | LOC101251314 | Chromosome 8 | 65666522 | 65667835 |
|  |  |  | MYBPA-2 | LOC101264349 | Chromosome 8 | 2867410 | 2869221 |
|  |  | MYB5 type | MYB5-1 | LOC101243923 | Chromosome 1 | 460031 | 461982 |
|  | *Nicotiana*  *tabacum* | TT2 type | TT2-1 | LOC107769603 | NW_015889608.1 | 67094 | 68666 |
|  |  |  | TT2-2 | LOC107773379 | NW_015897844.1 | 230 | 1997 |
|  |  | MYB5 type | MYB5-1 | LOC107807473 | NW_015944592.1 | 80192 | 82387 |
|  |  |  | MYB5-2 | LOC107776543 | NW_015901899.1 | 23660 | 25625 |
|  |  | MYBPA type | MYBPA-1 | LOC107766557 | NW_015886688.1 | 65276 | 66901 |
|  |  |  | MYBPA-2 | LOC107823382 | NW_015834182.1 | 152074 | 154432 |
|  |  |  | MYBPA-3 | LOC107759231 | NW_015787837.1 | 116820 | 118334 |
|  |  |  | MYBPA-4 | LOC107799919 | NW_015931887.1 | 116576 | 118873 |
|  | *Punica*  *granatum* | TT2 type | TT2 | LOC116192926 | Chromosome 1 | 14981445 | 14982852 |
|  |  | MYBPA type | MYBPA-1 | LOC116193704 | Chromosome 1 | 50277448 | 50279233 |
|  |  |  | MYBPA-2 | LOC116205321 | Chromosome 4 | 7828142 | 7829886 |
|  |  | MYB5 type | MYB5-1 | LOC116210714 | Chromosome 6 | 24439983 | 24442024 |
|  |  |  | MYB5-2 | LOC116205201 | Chromosome 4 | 34033661 | 34035738 |
|  | *Eucalyptus*  *grandis* | TT2 type | TT2 | LOC104438144 | Chromosome 8 | 54508865 | 54510233 |
|  |  | MYBPA type | MYBPA-1 | LOC104442190 | Chromosome 4 | 36921563 | 36923422 |
|  |  |  | MYBPA-2 | LOC104444093 | Chromosome 11 | 37672593 | 3767431 |
|  |  |  | MYBPA-3 | LOC104441632 | Chromosome 4 | 28219323 | 28222431 |
|  |  | MYB5 type | MYB5-1 | LOC104433774 | Chromosome 2 | 54096948 | 54098772 |
|  |  |  | MYB5-2 | LOC104443016 | Chromosome 1 | 40906790 | 40908635 |
|  |  |  | MYB5-3 | LOC104425277 | Chromosome 11 | 19450596 | 19452343 |
|  | *Populus*  *trichocarpa* | TT2 type | TT2-1 | LOC7491994 | chromosome 6 | 22727346 | 22728838 |
|  |  |  | TT2-2 | LOC18107616 | Chromosome 18 | 4589730 | 4591294 |
|  |  |  | TT2-3 | LOC127904682 | Chromosome 18 | 4709028 | 4710500 |
|  |  |  | TT2-4 | LOC7476671 | Chromosome 18 | 4439088 | 4442692 |
|  |  | MYBPA type | MYBPA-1 | LOC7497264 | Chromosome 2 | 13399634 | 13401571 |
|  |  |  | MYBPA-2 | LOC7486782 | Chromosome 14 | 6657097 | 6659274 |
|  |  | MYB5 type | MYB5-1 | LOC7483247 | Chromosome 1 | 343428 | 345213 |
|  |  |  | MYB5-2 | LOC7463575 | Chromosome 3 | 21431354 | 21433120 |
|  |  |  | MYB5-3 | LOC7489662 | Chromosome 13 | 4106882 | 4108890 |
|  |  |  | MYB5-4 | LOC7469886 | Chromosome 19 | 4904130 | 4957688 |
|  |  |  | MYB5-5 | LOC18104119 | Chromosome 13 | 4124880 | 4126831 |
|  | *Manihot*  *esculenta* | TT2 type | TT2-1 | LOC110603359 | Chromosome 16 | 29158331 | 29159478 |
|  |  |  | TT2-2 | LOC110610671 | Chromosome 3 | 4922841 | 4924305 |
|  |  | MYBPA type | MYBPA-1 | LOC110615013 | Chromosome 5 | 1880463 | 1881707 |
|  |  |  | MYBPA-2 | LOC110609171 | Chromosome 2 | 605573 | 60703 |
|  |  | MYB5 type | MYB5-1 | LOC110601742 | Chromosome 15 | 8665150 | 8666778 |
|  |  |  | MYB5-2 | LOC110620710 | Chromosome 8 | 38710802 | 38713447 |
|  |  |  | MYB5-3 | LOC110623638 | Chromosome 9 | 33900938 | 33902737 |
|  | *Juglans*  *regia* | TT2 type | TT2-1 | LOC109008735 | Chromosome 1 | 35164369 | 35165884 |
|  |  |  | TT2-2 | LOC109001985 | Chromosome 7 | 43344877 | 43346536 |
|  |  |  | TT2-3 | LOC109019805 | Chromosome 12 | 26044441 | 26047561 |
|  |  |  | TT2-4 | LOC109019804 | Chromosome 12 | 26062099 | 26069432 |
|  |  |  | TT2-5 | LOC109008728 | Chromosome 1 | 35093663 | 35095275 |
|  |  |  | TT2-6 | LOC109006176 | Chromosome 8 | 3213894 | 3215272 |
|  |  |  | TT2-7 | LOC109008746 | Chromosome 1 | 35268085 | 35270469 |
|  |  |  | TT2-8 | LOC109008699 | Chromosome 1 | 34956472 | 34958210 |
|  |  | MYBPA type | MYBPA1 | LOC109010374 | Chromosome 12 | 7137355 | 7138876 |
|  |  |  | MYBPA2 | LOC108989285 | Chromosome 7 | 11254553 | 11256367 |
|  |  |  | MYBPA3 | LOC109002060 | Chromosome 2 | 28795095 | 28796321 |
|  |  |  | MYBPA4 | LOC108993750 | Chromosome 9 | 18759648 | 18760921 |
|  |  | MYB5 type | MYB5 | LOC108982488 | Chromosome 5 | 22247197 | 22249166 |
|  | *Quercus*  *variabilis* | TT2 type | TT2-1 | LOC112011853 | NW_019826308.1 | 433741 | 435197 |
|  |  |  | TT2-2 | LOC112003598 | NW_019823491.1 | 7147 | 8793 |
|  |  |  | TT2-3 | LOC112010882 | NW_019807288.1 | 17400 | 19052 |
|  |  |  | TT2-4 | LOC112023509 | NW_019809575.1 | 464390 | 468712 |
|  |  | MYBPA type | MYBPA1 | LOC112035506 | NW_019814393.1 | 46583 | 48422 |
|  |  |  | MYBPA2 | LOC112022241 | NW_019809027.1 | 103718 | 104989 |
|  |  | MYB5 type | MYB5-1 | LOC112004729 | NW_019824103.1 | 286109 | 288064 |
|  |  |  | MYB5-2 | LOC112003883 | NW_019823610.1 | 401349 | 402563 |
|  | *Fragaria*  *vesca* | TT2 type | TT2-1 | LOC101301599 | LG6 | 20898671 | 20899921 |
|  |  |  | TT2-2 | LOC101296688 | LG6 | 24345878 | 24347786 |
|  |  | MYBPA type | MYBPA-1 | LOC101306459 | LG7 | 3193391 | 3194814 |
|  |  |  | MYBPA-2 | LOC101312063 | LG5 | 8379156 | 8380281 |
|  |  |  | MYBPA-3 | LOC101309064 | LG5 | 25231844 | 25232788 |
|  |  | MYB5 type | MYB5 | LOC101308850 | LG3 | 24004054 | 24005966 |
|  | *Glycine*  *max* | TT2 type | TT2-1 | LOC100781908 | Chromosome 13 | 21330671 | 21332327 |
|  |  |  | TT2-2 | LOC100809225 | Chromosome 16 | 594664 | 596527 |
|  |  | MYBPA type | MYBPA-1 | LOC100803345 | Chromosome 7 | 4697595 | 4699092 |
|  |  |  | MYBPA-2 | [LOC100101857](https://www.ncbi.nlm.nih.gov/gene/100101857) | Chromosome 16 | 2169882 | 2171174 |
|  |  | MYB5 type | MYB5-1 | [LOC100782308](https://www.ncbi.nlm.nih.gov/gene/100782308) | Chromosome 14 | 29482128 | 29483382 |
|  |  |  | MYB5-2 | LOC100797847 | Chromosome 13 | 9402399 | 9404621 |
|  | *Brassica*  *napus* | TT2 type | TT2-1 | LOC106359996 | Chromosome C8 | 15666481 | 15667607 |
|  |  |  | TT2-2 | LOC106418890 | Chromosome A8 | 10864265 | 10865366 |
|  |  | MYB5 type | MYB5-1 | LOC106444546 | Chromosome C5 | 38579798 | 38582227 |
|  |  |  | MYB5-2 | LOC106347585 | Chromosome A1 | 31080529 | 31083899 |
|  |  |  | MYB5-3 | LOC106345394 | Chromosome A5 | 28406735 | 28408811 |
|  |  |  | MYB5-4 | LOC106377439 | Chromosome C1 | 48771518 | 48774741 |
|  | *Arabidopsis*  *thaliana* | TT2 type | TT2 | AT5G35550 | Chromosome 5 | 13726743 | 13727961 |
|  |  | MYB5 type | MYB5 | AT3G13540 | Chromosome 3 | 4419960 | 4421751 |
|  | *Coptis*  *chinensis* | TT2 type | TT2-1 | IFM89_026489 | chromosome 3 | 103745678 | 103746885 |
|  |  |  | TT2-2 | IFM89_036822 | chromosome 9 | 6709799 | 6710767 |
|  |  | MYB5 type | MYB5-1 | IFM89_012293 | chromosome 7 | 7854188 | 7855051 |
|  |  |  | MYB5-2 | IFM89_012294 | chromosome 7 | 7874913 | 7876422 |
| Monoco  tyledons | *Oryza*  *sativa* | TT2 type | TT2 | LOC4333088 | Chromosome 3 | 16879301 | 16883742 |
|  |  | MYB5 type | MYB5 | LOC4323897 | Chromosome 1 | 28796372 | 28797741 |
|  | *Sorghum*  *bicolor* | TT2 type | TT2 | LOC8080419 | Chromosome 1 | 62819031 | 62822538 |
|  |  | MYB5 type | MYB5 | LOC8068060 | Chromosome 3 | 60404207 | 60405627 |
|  | *Zea mays* | TT2 type | TT2 | LOC732799 | Chromosome 6 | 119938927 | 119940495 |
|  |  | MYB5 type | MYB5 | LOC103636205 | Chromosome 8 | 156716267 | 156717499 |
|  | *Ananas*  *comosus* | TT2 type | TT2-1 | LOC109704317 | NW_017890836.1 | 13637 | 15617 |
|  |  |  | TT2-2 | LOC109719652 | NC_033621.1 | 1923896 | 1925950 |
|  |  |  | TT2-3 | LOC109706575 | NC_033622.1 | 11425277 | 11426525 |
|  |  |  | TT2-4 | LOC109706592 | NC_033622.1 | 11286474 | 11287673 |
|  |  |  | TT2-5 | LOC109706352 | NW_017893575.1 | 47762 | 49333 |
|  |  | MYB5 type | MYB5 | LOC109716607 | NC_033630.1 | 1851239 | 1852634 |
| Basal  angiosperms | *Nymphaea*  *colorata* | MYB5 type | MYB5 | LOC116256740 | Chromosome 6 | 20807439 | 20809203 |
|  | *Amborella*  *trichopoda* | TT2 type | TT2-1 | LOC18439413 | NW_006499705.1 | 4638125 | 4640007 |
|  |  |  | TT2-2 | LOC18439414 | NW_006499705.1 | 4655687 | 4658558 |
|  |  |  | TT2-3 | LOC18439415 | NW_006499705.1 | 4670495 | 4672421 |
|  |  | MYB5 type | MYB5-1 | LOC18448829 | NW_006500356.1 | 1163996 | 1165721 |
|  |  |  | MYB5-2 | LOC18448830 | NW_006500356.1 | 1205229 | 1206712 |
|  |  |  | MYB5-3 | LOC18448827 | NW_006500356.1 | 1094496 | 1095914 |
| gymnosperms | *Ginkgo biloba* | TT2 type | NA |  |  |  |  |
|  |  | MYBPA type | NA |  |  |  |  |
|  |  | MYB5 type | NA |  |  |  |  |
| pteridophytes | *Alsophilo spinulosa* | TT2 type | NA |  |  |  |  |
|  |  | MYBPA type | NA |  |  |  |  |
|  |  | MYB5 type | NA |  |  |  |  |
| mosses | *Anthoceros angustus* | TT2 type | NA |  |  |  |  |
|  |  | MYBPA type | NA |  |  |  |  |
|  |  | MYB5 type | NA |  |  |  |  |
